# Positive Role of Promyelocytic Leukemia Protein in Type I Interferon Response and Its Regulation by Human Cytomegalovirus

**DOI:** 10.1371/journal.ppat.1004785

**Published:** 2015-03-26

**Authors:** Young-Eui Kim, Jin-Hyun Ahn

**Affiliations:** Department of Molecular Cell Biology, Samsung Biomedical Research Institute, Sungkyunkwan University School of Medicine, Suwon, Republic of Korea; University of Glasgow, UNITED KINGDOM

## Abstract

Promyelocytic leukemia protein (PML), a major component of PML nuclear bodies (also known as nuclear domain 10), is involved in diverse cellular processes such as cell proliferation, apoptosis, gene regulation, and DNA damage response. PML also acts as a restriction factor that suppresses incoming viral genomes, therefore playing an important role in intrinsic defense. Here, we show that PML positively regulates type I interferon response by promoting transcription of interferon-stimulated genes (ISGs) and that this regulation by PML is counteracted by human cytomegalovirus (HCMV) IE1 protein. Small hairpin RNA-mediated PML knockdown in human fibroblasts reduced ISG induction by treatment of interferon-β or infection with UV-inactivated HCMV. PML was required for accumulation of activated STAT1 and STAT2, interacted with them and HDAC1 and HDAC2, and was associated with ISG promoters after HCMV infection. During HCMV infection, viral IE1 protein interacted with PML, STAT1, STAT2, and HDACs. Analysis of IE1 mutant viruses revealed that, in addition to the STAT2-binding domain, the PML-binding domain of IE1 was necessary for suppression of interferon-β-mediated ISG transcription, and that IE1 inhibited ISG transcription by sequestering interferon-stimulated gene factor 3 (ISGF3) in a manner requiring its binding of PML and STAT2, but not of HDACs. In conclusion, our results demonstrate that PML participates in type I interferon-induced ISG expression by regulating ISGF3, and that this regulation by PML is counteracted by HCMV IE1, highlighting a widely shared viral strategy targeting PML to evade intrinsic and innate defense mechanisms.

## Author Summary

For productive viral infection, virus needs to overcome successive host defenses including intrinsic defense and innate and acquired immunity. Promyelocytic leukemia protein (PML) has been shown to play an important role in intrinsic defense by acting as a nuclear restriction factor that suppresses incoming viral genomes. In this study, we demonstrate that PML also positively regulates type I interferon response by promoting transcription of interferon-stimulated genes (ISGs). Therefore, PML is a key player in both intrinsic and innate host defenses. We further show that this regulation by PML in type I interferon response is inhibited by human cytomegalovirus (HCMV) IE1 protein, which forms a complex with PML, STAT1, STAT2, and HDACs in virus-infected cells. By analyzing mutant viruses, we demonstrate that IE1 inhibits ISG transcription by sequestering interferon-stimulated gene factor 3 (ISGF3) in a manner requiring its binding of PML and STAT2, but not of HDACs. Our findings reveal that PML is a regulator of ISGF3 in type I interferon response and that this PML activity is counteracted by HCMV IE1. Our study explains why PML targeting activity is widely conserved among many viruses.

## Introduction

Type I interferons (IFNs) are multifunctional cytokines that act as key components of innate immune response to viral infection. Virus infections rapidly trigger induction of IFNα and/or IFNβ through activating nuclear factor-kappa B (NF-κB) and interferon regulatory factor 3 (IRF3) transcription factors. The binding of newly synthesized IFNα and/or IFNβ to their receptors leads to tyrosine phosphorylation of cytoplasmic signal transducers and activators of transcription (STAT1 and STAT2) via Janus kinase 1 (Jak1). Phosphorylated STAT1 and STAT2 heterodimerize and rapidly translocate to the nucleus, where they assemble with IFN regulatory factor 9 (IRF9) to form a transcription complex known as IFN-stimulated gene factor 3 (ISGF3), which sequence-specifically binds to an IFN-stimulated response element (ISRE) present in type I IFN-stimulated genes (ISGs), many of which exhibit antiviral activity [1]. ISGF3 specifically interacts with several coactivators including histone acetyltransferases (HATs) [2, 3], histone deacetylases (HDACs) [4–7], and nucleosome remodeling factors [8].

Promyelocytic leukemia protein (PML), also named TRIM19, belongs to the tripartite motif family (TRIM) of proteins that contain a RING finger, two B-boxes, and an α-helical coiled-coil (RBCC) domain [9, 10]. As a major component of PML nuclear bodies (NBs) (also known as nuclear domain 10) [11], PML is involved in diverse cellular processes, including proliferation, apoptosis, gene transcription, and DNA damage response [12–14]. PML expression is increased by IFNs [15, 16]. Various PML isoforms are expressed via alternative splicing by sharing the same amino terminus [10, 17]. PML and other major components of PML NBs, such as Sp100, Daxx, and ATRX, exhibit antiviral activities as nuclear intrinsic restriction factors that suppress incoming viral genomes [18–21].

Many viruses encode proteins that interfere with the antiviral activity of PML and most research has focused on the viral countermeasures against the antiviral activity of PML as an intrinsic restriction factor that recognizes incoming viral genomes and suppresses the initiation of viral gene expression [18, 21]. The most widely studied example is ICP0 protein of herpes simplex virus type-1 (HSV-1). ICP0 acts as a ubiquitin E3 ligase that preferentially targets the SUMO-modified PML isoforms, leading to their degradation [22]. In human cytomegalovirus (HCMV) infections, immediate-early (IE) 1 protein interacts with PML and disrupts PML NBs [23–26]. This activity of IE1 correlates with the functional activities of IE1 during infection and the antiviral role of PML in HCMV replication is well established using PML-overexpressing and knockdown cells [27–30]. We previously demonstrated that the central hydrophobic region of IE1 is required for the activities of IE1 to bind PML and induce PML deSUMOylation leading to PML NB disruption and to transactivate several viral and cellular promoters [28, 31]. IE1 also interacts with STAT2 and to a lesser extent with STAT1, and promotes efficient viral growth by down-regulating type I IFN signaling [32–34]. The IE1-STAT2 interaction requires the near C-terminal region of IE1 including its acidic domain [33–35]. Recently, the crystal structure of the central hydrophobic region of IE1, named IE1core, has been solved and the data show that IE1core shares secondary structure features with the coiled-coil domain of TRIM proteins [36]. IE1 has been shown to interact with HDAC3 [37].

Recently, a new role of PML in IFNγ-induced gene expression has been demonstrated. In PML^-/-^ mouse embryonic fibroblasts and PML-depleted cells, INFγ-induced phosphorylation of STAT1 and its binding to the promoters of ISGs were diminished [38]. PML was also shown to enhance IFNγ-induced MHC class II gene expression by targeting class II transactivator (CIITA) and preventing its degradation [39]. In addition, a specific PML isoform was shown to participate in the production of IFNβ [40]. However, the role of PML in type I interferon response is not fully understood.

In this study, we show that PML participates in type I IFN-induced ISG expression by regulating ISGF3 and that this regulation by PML is counteracted by HCMV IE1. Our results reveal a new role of PML in type I IFN response and highlight a widely shared viral strategy of targeting PML to evade both host intrinsic and innate defense mechanisms.

## Results

### PML positively regulates ISG expression

**Fig 1:**
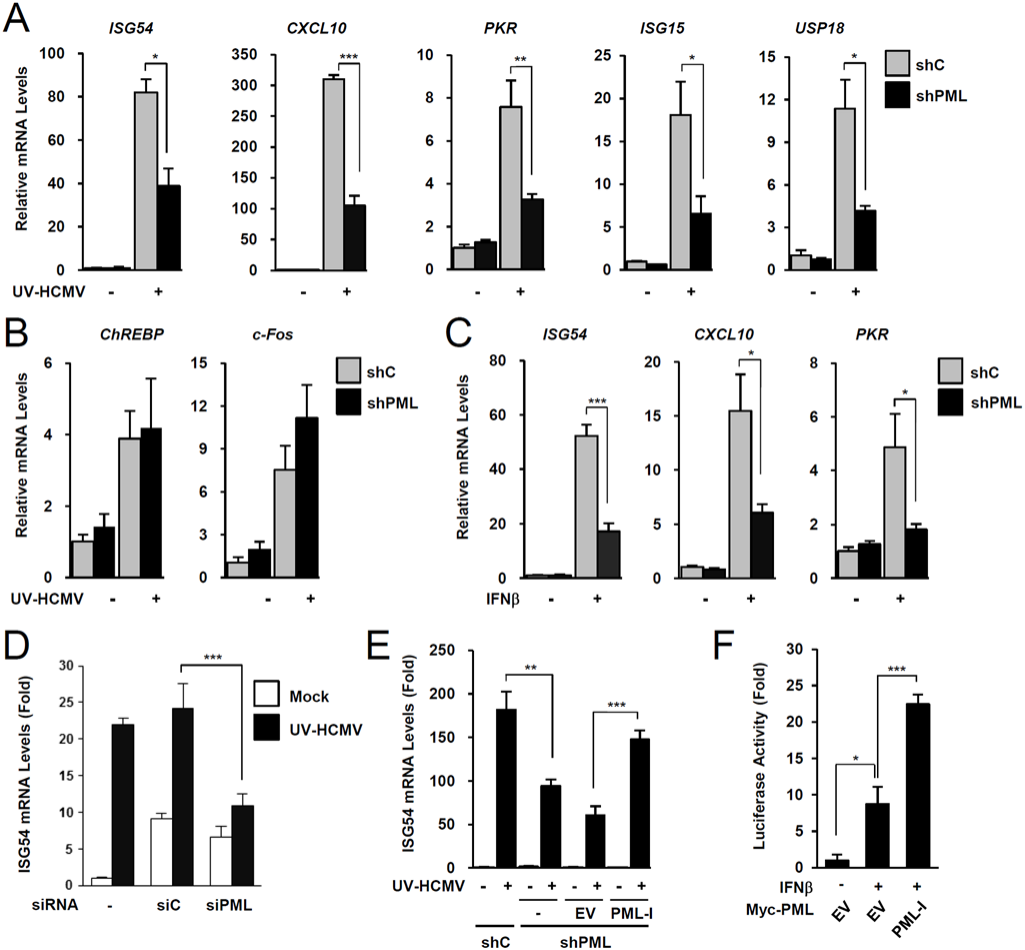
Effects of PML knockdown on ISG induction by IFNβ treatment or UV-HCMV infection. (A-C) Control (shC) and PML-knockdown (shPML) HF cells (see S1A and S1B Figs) were mock-infected or infected with UV-HCMV at an MOI of 5 IFU (infectious units) per cell (A and B), or untreated or treated with IFNβ (1 x 10^3^ units/ml) (C), for 8 h. Total RNAs were prepared and ISG54, CXCL10, and PKR mRNA were quantified by qRT-PCR. The amounts of mRNA in cells treated with INFβ or infected with UV-HCMV over those of untreated or mock-infected cells are presented as fold inductions. The results shown are the mean values and standard errors of at least three independent experiments. (D) HF cells were transfected with 20 nmole of control scrambled (siC) or PML-specific siRNA (siPML) twice at times 0 and 24 h. At 72 h, cells were mock-infected or infected with UV-HCMV and ISG54 mRNA levels were measured by qRT-PCR as described in (A). PML-knockdown was confirmed by immunoblotting (see S1C Fig). (E) shC and shPML HF cells transduced with empty retroviral vector or PML-I-expressing vector were mock-infected or infected with UV-HCMV and ISG54 mRNA levels were determined by qRT-PCR as described in (A). The amounts of mRNA in cells infected with UV-HCMV over those in mock-infected cells are presented as fold inductions. Refer to S1D Fig for the expression levels of PML-I in shPML cells. (F) shPML HF cells were cotransfected with 0.5 μg of the ISG54 ISRE-Luc reporter plasmid and 1 μg of empty vector or plasmid encoding myc-PML-I as indicated. At 24 h, cells were untreated or treated with IFNβ (1 x 10^3^ units/ml) for 8 h, and luciferase reporter assays were performed. See S1E Fig for the expression levels of PML proteins.

To investigate the role of PML in type I IFN signaling, control and PML-knockdown HF cells were produced using retroviral vectors expressing control (shC) or PML-specific shRNA (shPML) (S1A and S1B Fig). When shC and shPML HF cells were infected with UV-inactivated HCMV (UV-HCMV), the mRNA levels of ISGs, such as ISG54, CXCL10, PKR, ISG15, and USP18 were less efficiently induced in PML-knockdown cells than in control cells (Fig. 1A). This effect of PML knockdown was specific for ISGs because PML knockdown did not affect the UV-HCMV-mediated induction of ChREBP, a glucose-responsive transcription factor known to be induced by HCMV infection [41], and c-Fos, a cellular immediate-early response gene (Fig. 1B). A similar effect of PML knockdown on IFNβ-mediated ISG transcription was observed in HF cells (Fig. 1C) and in HEK 293 cells (S2A Fig). Transient transfection of HF cells with control siRNA (siC) or PML-specific siRNA (siPML), which targeted different sequences from those targeted by shPML, moderately increased ISG54 transcription probably via dsRNA sensing [42]. However, when siRNA transfected cells were infected with UV-HCMV, ISG54 transcription was less effectively induced in siPML transfected cells than in siC transfected cells (Figs. 1D and S1C). Collectively, these results indicate that PML depletion reduces the IFNβ or UV-HCMV-mediated induction of ISG transcription.

We also investigated whether re-expression of PML reverses the effect of PML depletion on ISG expression. When shPML HF cells (Puro^+^) were transduced by retroviral vectors (Neo^+^) expressing PML-I (the most abundant PML isoform), whose target sequences for shPML were mutated to confer resistance, the re-expression of PML-I partially reversed the effect of PML knockdown (Figs. 1E and S1D). Furthermore, transfection of this PML-I into PML-depleted HF cells increased IFNβ-mediated ISG54 promoter activation in reporter assays (Figs. 1F and S1E). These results indicate that PML is required for efficient type I IFN-mediated ISG transcription after IFNβ treatment or HCMV infection.

### PML promotes accumulation of activated STAT1 and STAT2

**Fig 2:**
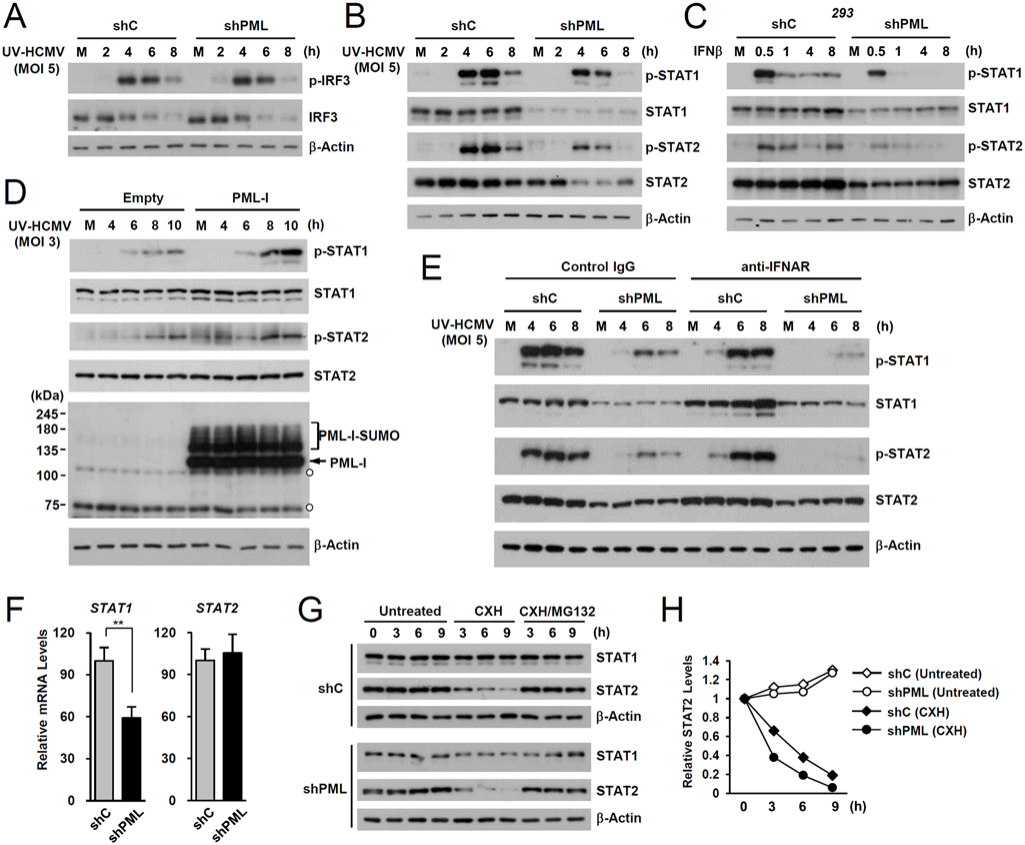
Effects of PML knockdown on the levels of STAT1 and STAT2 and of their activated forms. (A) shC and shPML HF cells were mock-infected (M) or infected with UV-HCMV at an MOI of 5 IFU per cell. Total cell lysates were prepared at the indicated time points and immunoblot analysis was performed with antibodies specific for IRF3 and its phosphorylated form. The level of β-actin was used as a loading control. (B and C) shC and shPML HF cells were uninfected or infected with UV-HCMV as in (A) (B), or shC and shPML 293 cells were untreated or treated with IFNβ (1 x 10^3^ units/ml) (C). Total cell lysates were prepared at the indicated time points and immunoblot analysis was performed with antibodies specific for STAT1, STAT2, and their phosphorylated forms. (D) shPML HF cells transduced with empty retroviral vectors or retroviral vectors expressing PML-I were mock-infected or infected with UV-HCMV at an MOI of 3 for the indicated times. Total cell lysates were prepared and immunoblotted using antibodies specific for PML (PG-M3), STAT1, STAT2, and their activated forms. (E) shC and shPML HF cells were uninfected or infected with UV-HCMV and immunoblotted as in (B), except that the culture medium added after virus adsorption included anti-IFNAR antibody or control IgG (5 μg/ml) as indicated. (F) STAT1 and STAT2 mRNA levels in shC and shPML HF cells were measured by qRT-PCR. (G) shC and shPML HF cells were untreated or treated with cycloheximide (CXH) (200 μg/ml) for the indicated times. Total cell lysates were prepared and immunoblotted. (H) Plots of STAT2 levels in cycloheximide treated or untreated shC and shPML HF cells.

To investigate the mechanism by which PML promotes ISG transcription, we compared levels of IRF3, STAT1, STAT2, and their activated forms in control and PML-knockdown HF cells after UV-HCMV infection. The activation of IRF3 after virus infection was comparable in control and PML-knockdown HF cells (Fig. 2A). However, levels of STAT1 and its activated form (p-STAT1) were significantly lower in PML-knockdown cells than in control cells (Fig. 2B). The level of activated STAT2 (p-STAT2) was also reduced by PML-knockdown, although the level of total STAT2 was only slightly reduced by PML depletion (Fig. 2B). A similar effect of PML knockdown on accumulation of STAT1, STAT2, and their activated forms was observed in 293 cells treated with IFNβ (Fig. 2C). Furthermore, the re-expression of PML-I in PML-depleted HF cells slightly increased total STAT1 and resulted in higher levels of the activated forms of STAT1 and STAT2 than in control cells (Fig. 2D). These results suggest that PML positively regulates the expression of STAT1 and the accumulation of activated forms of STAT1 and STAT2.

To investigate whether the effect of PML on the expression of STAT1 and STAT2 shown in Fig. 2B involves an indirect consequence of type I IFNs, we compared the effects of PML knockdown on STAT1 and STAT2 activation after treatment of HF cells with control IgG or anti-IFNα/β receptor (IFNAR) antibody. The results showed that in both control (shC) and PML-knockdown (shPML) cells, anti-IFNAR antibody treatment moderately reduced (or delayed) activation of STAT1 and STAT2, indicating involvement of an indirect effect of type I IFNs on activation of STAT proteins in this assay. However, the results also showed that in anti-IFNAR antibody-treated cells, PML knockdown still significantly reduced the expression of both unmodified and phosphorylated forms of STAT1 and STAT2 compared to control shC cells. This result demonstrates that PML primarily affects STAT1 and STAT2 expression, although it can partly affect the activation of STAT proteins via an autoregulatory loop (Fig. 2E).

We next investigated the effect of PML knockdown on the mRNA levels of STAT1 and STAT2. In qRT-PCR analysis, transcription level of STAT1 in PML-knockdown HF was decreased to 60% of control cells, while STAT2 transcription was unaffected (Fig. 2F). A similar effect of PML knockdown on the mRNA level of STAT1 was observed in 293 cells (S2B Fig). These results indicate that PML is required for efficient accumulation of STAT1 transcripts. Analysis of protein stability using cycloheximide treatment showed that, unlike STAT1, STAT2 was rapidly degraded by proteasomes in HF cells, and that PML depletion shortened the half-life of STAT2 (Fig. 2G and 2H). These results demonstrate that PML facilitates the transcription of STAT1 and the stable expression of STAT2 protein.

### PML associates with STAT1, STAT2, HDAC1, and HDAC2 and with ISG promoters

**Fig 3:**
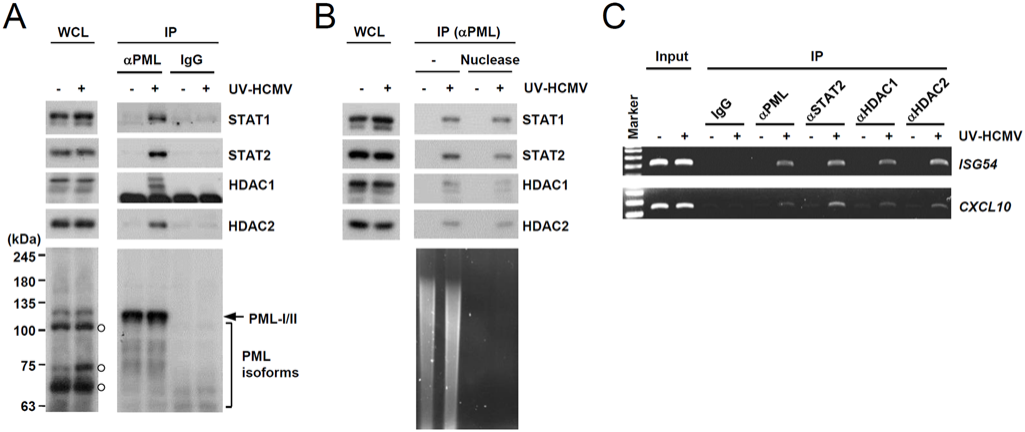
Association of PML with STAT1, STAT2, and HDAC1 on ISG54 and CXCL10 promoters after UV-HCMV infection. (A) Normal HF cells were mock-infected (-) or infected with UV-HCMV (+) at an MOI of 3 IFU per cell for 8 h and co-IP assays were carried out. Total cell lysates were prepared and immunoprecipitated with anti-PML antibody (PG-M3) or mouse IgG as a negative control. Immunoprecipitated samples and whole cell lysates were subjected to SDS-PAGE and then immunoblotted with antibodies for STAT1, STAT2, HDAC1, HDAC2, and PML (PG-M3). (B) Co-IP assays were performed as described in (A) using cell lysates treated with nucleases. (C) HF cells were infected as described in (A) and ChIP assays were performed using anti-PML (PG-M3), anti-STAT2, anti-HDAC1, and anti-HDAC2 antibodies. PCR was performed to detect ISG54 and CXCL10 promoter DNAs. The sizes of the DNA fragments amplified from the ISG54 and CXCL10 promoter regions were 199 bp and 241 bp, respectively. A 100 bp DNA ladder was used as a size marker.

We next investigated whether PML interacts with STAT1 and STAT2 components of ISGF3. Since PML directly interacts with HDAC1 and HDAC2 [43], which may affect ISG transcription, we also assessed whether PML associates with these HDACs after UV-HCMV infection. In co-IP assays performed at 8 h after infection, PML was found to interact with STAT1, STAT2, HDAC1, and HDAC2 in UV-HCMV-infected cells but not in uninfected cells (Fig. 3A). These interactions did not involve DNA, since a similar association was detected in cell lysates treated with nucleases (Fig. 3B). These interactions of PML with STAT1, STAT2, HDAC1, and HDAC2, but not with IRF9 and ribonucleotide reductase R1 (as negative controls), were also observed in cells treated with IFNβ (S3A Fig). Furthermore, in ChIP assays, PML was found to associate with ISG54 and CXCL10 promoters in UV-HCMV-infected or IFNβ-treated cells but not in mock-infected cells, as was also observed for STAT2, HDAC1, and HDAC2 (Figs. 3C and S3B). These results demonstrate that the associations of PML with STAT1, STAT2, HDAC1, and HDAC2 and with ISG promoters are induced after UV-HCMV infection. Therefore, PML not only promotes the accumulation of activated STAT1 and STAT2, but also affects ISG expression by directly associating with ISGF3 and HDACs.

### PML binding by HCMV IE1 is required for the efficient repression of INFβ-induced ISG expression

**Fig 4:**
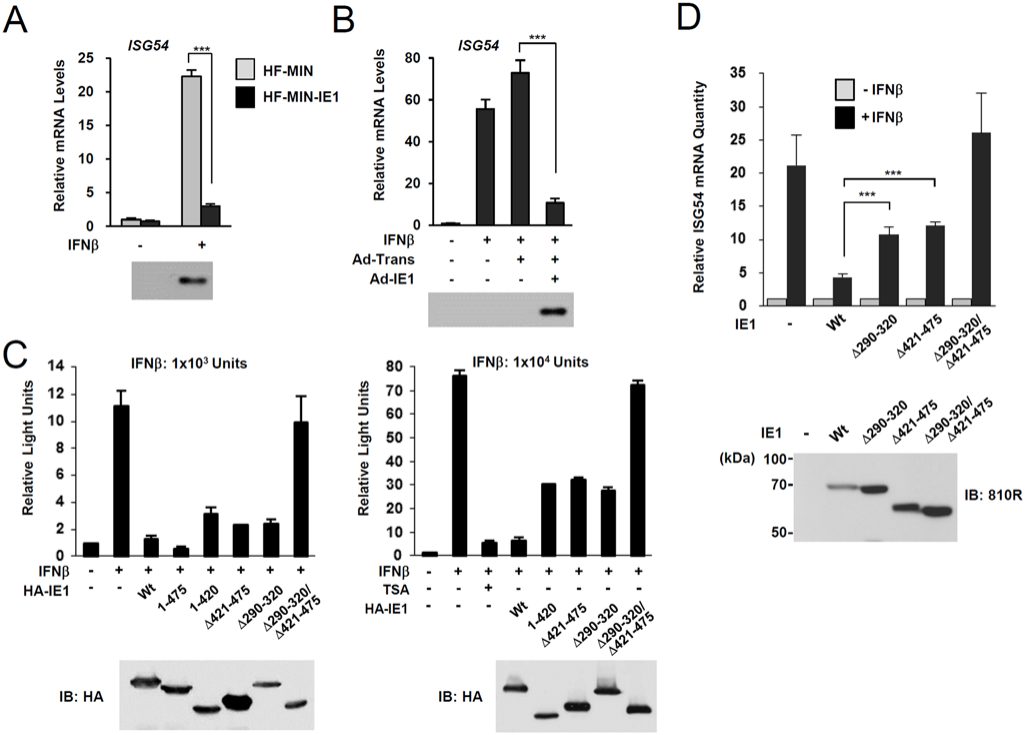
Effects of ectopic IE1 expression on the IFNβ-mediated ISG54 induction. (A) HF cells transduced by control MIN retroviral vector (HF-MIN) or MIN-IE1 (HF-MIN-IE1) were untreated or treated with IFNβ (1 x 10^3^ units/ml) for 8 h. Total RNAs were prepared and the mRNA levels of ISG54 and β-actin were determined by qRT-PCR. The IE1 protein levels in cells were determined by immunoblotting with anti-IE1 antibody (6E1). (B) HF cells were uninfected or infected with Ad-Trans alone or with Ad-Trans plus Ad-IE1 at a total MOI of 10 plaque forming units (PFU) per cell for 48 h. Cells were then incubated for 8 h in the absence or presence of IFNβ (1 x 10^3^ units/ml). qRT-PCR and immunoblot assays were performed as described in (A). (C) Reporter assays using the ISG54 ISRE-luciferase (Luc) reporter construct. 293T cells were cotransfected with 0.5 μg of ISG54 ISRE-Luc reporter plasmid and 0.5 μg of plasmid encoding intact HA-IE1, HA-IE1(1–475), or HA-IE1(Δ421–475) or 1.5 μg of plasmid encoding HA-IE1(Δ290–320), HA-IE1(1–420), or HA-IE1(Δ290–320/Δ421–475) mutants as indicated. All samples for transfection were made up to the same total amount of DNA using empty vectors. At 24 h, cells were untreated or treated with 1 x 10^3^ units/ml (left) or 1 x 10^4^ units/ml of IFNβ in absence or presence of 1 μM tricostatin A (TSA) (right) for 8 h, and luciferase reporter assays were performed. The results shown are the mean values and standard errors of three independent experiments. The expression levels of IE1 proteins were determined by immunoblotting with anti-HA antibody. (D) HF cells stably expressing wild-type or mutant IE1 (produced using retroviral vectors) were untreated or treated with IFNβ (1 x 10^3^ units/ml) for 8 h. Total RNAs were prepared and the amounts of ISG54 mRNA were determined by qRT-PCR. The results shown are the mean values and standard errors of three independent experiments. The amounts of mRNA in cells treated with IFNβ compared to that in untreated cells are shown as fold inductions. The expression levels of IE1 were determined by immunoblotting using anti-IE1/IE2 antibody (810R).

During HCMV infection, IE1 effectively inhibits IFNβ-induced ISG expression [32–34]. Consistently, IE1 expression in HF cells by retroviral vectors or adenoviral vectors efficiently blocked IFNβ-induced ISG54 expression at transcription level (Fig. 4A and 4B). To determine whether PML binding by IE1 contributes to the ability of IE1 to inhibit ISG transcription, we examined the effects of wild-type and mutant IE1 on ISG expression by performing reporter assays in permissive HF cells using the ISG54 ISRE-luciferase reporter construct. IE1 expression or treatment with trichostatin A (TSA; a general HDAC inhibitor) repressed the IFNβ-induced activation of this ISRE-containing promoter. However, the expression of IE1(Δ290–320), which was defective in binding PML [28], or IE1(Δ421–475), which was defective in binding STAT2 [33, 35], less effectively inhibited ISRE promoter than wild-type IE1 and this was more evident when a higher dose of IFNβ was used (Fig. 4C). IE1(Δ290–320/Δ421–475), in which the regions required for binding PML and STAT2 are deleted, had no activity in repressing ISRE activation (Fig. 4C). We also analyzed accumulation of ISG54 mRNA in HF cells stably expressing wild-type or mutant IE1 proteins. Consistent with the results of reporter gene assays, IE1(Δ290–320) and IE1(Δ421–475) less efficiently inhibited INFβ-induced ISG54 transcription than wild-type IE1, and IE1(Δ290–320/Δ421–475) completely lost the repressive activity (Fig. 4D). These results show that PML binding is required for IE1 to repress IFNβ-induced ISG transcription.

### PML-binding defective mutant virus does not efficiently repress ISG expression

**Fig 5:**
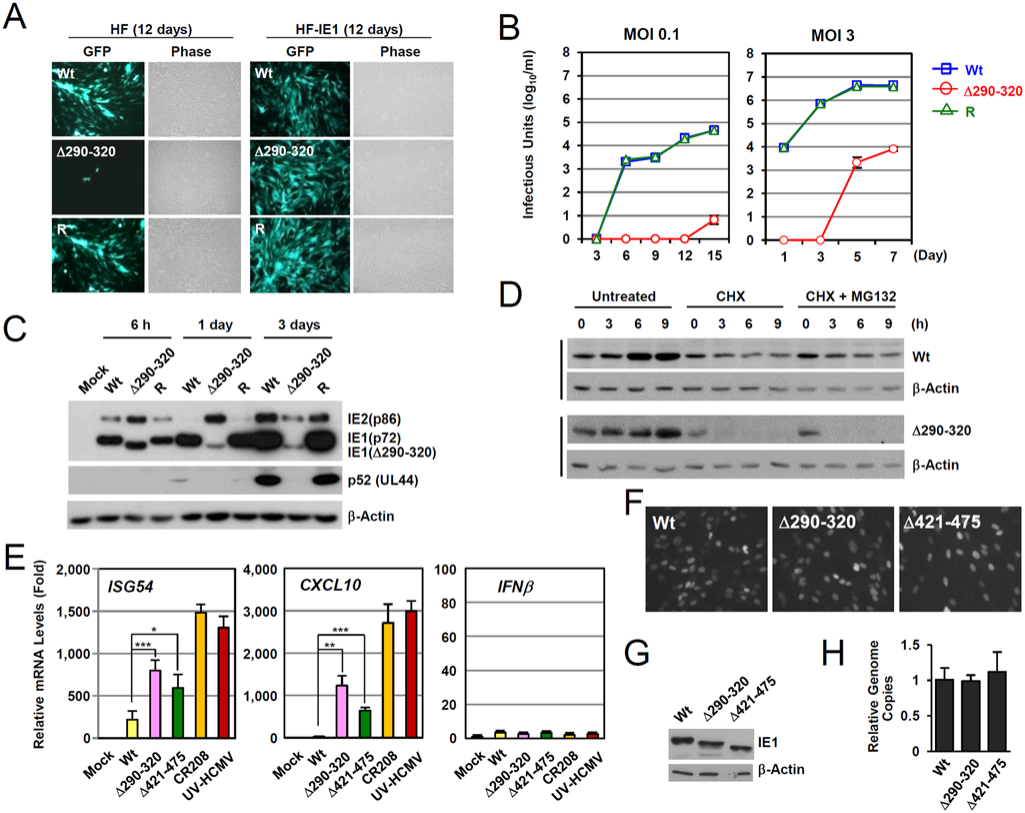
Growth properties of the mutant virus encoding IE1(Δ290–320). (A) HF and HF-IE1 cells were transfected via electroporation with T-BAC DNAs encoding wild-type IE1, IE1(Δ290–320) mutant, or its revertant. GFP images were taken at 12 days after transfection. (B) HF cells were infected with wild-type, IE1(Δ290–320) mutant, or revertant virus at an MOI of 0.1 or 3 IFU per cell. The growth curve shown represents the total number of infectious center-forming units in culture supernatants at the indicated sampling times. (C) HF cells were infected with recombinant viruses [wild-type (Wt), IE1(Δ290–320) mutant, or revertant (R)] at an MOI of 3. Total cell lysates were prepared at the indicated times, and immunoblotting was performed using anti-IE1/IE2 (810R), anti-p52, or anti-β-actin antibodies. (D) The stability of IE1(Δ290–320) in virus-infected cells. HF cells were infected with wild-type or IE1(Δ290–320) virus at an MOI of 3. At 6 h post-infection, cells were untreated or treated with 200 μl per ml of cycloheximide (CHX) or with 200 μl per ml of CHX plus 0.5 μM of MG132, and then further incubated for the indicated times. Cells were harvested and immunoblot assays were carried out using anti-IE1 (6E1) or anti- β-actin antibodies. β-Actin was used as a loading control. (E-H) Comparison of the effects of wild-type or mutant IE1expression on type I IFN response during virus infection. HF cells were mock-infected or infected with wild-type, IE1(Δ290–320), IE1(Δ421–475), and CR208 viruses, which were grown in IE1-expressing cells, or UV-HCMV at an MOI of 3. At 12 h post-infection, total RNAs were prepared and ISG54, CXCL10, or IFNβ mRNA levels were determined by qRT-PCR. The results shown are the mean values and standard errors of three independent experiments (E). The comparable expression of IE1 in recombinant virus-infected cells at 12 h after infection was shown by performing IFA (F) and immunoblotting (G) using two different anti-IE1 antibodies, 810R and 6E1, respectively. The genome copies of input viruses in cells immediately after virus adsorption (at time zero) were determined by qPCR (H).

The IE1(Δ290–320) mutant protein contains a 30 amino acid deletion within the central hydrophobic region, which has been shown to form highly ordered structures [36]. The HCMV(Towne)-BAC clone encoding IE1(Δ290–320) was not infectious when introduced into permissive HF cells, indicating that this 30-amino acid region is required for efficient viral growth [28]. To grow IE1(Δ290–320) mutant virus, HF cells expressing wild-type IE1 (HF-IE1) were produced by retroviral transduction, and these cells were found to support the growth of CR208 [44], an IE1 exon 4-deleted mutant virus (S4A Fig). Similar to wild-type and revertant viruses, the mutant virus expressing IE1(Δ290–320) was also grown to high titers in HF-IE1 cells (Fig. 5A). The IE1(Δ290–320) protein failed to disrupt PML NBs during infection (S4B Fig).

We next analyzed the multi-step and single-step growth curves of IE1(Δ290–320) virus in HF cells. At an MOI of 0.1 (IFU per cell), progeny mutant virions were undetectable until 12 days after infection. The mutant virus had 6,800-fold lower titers than those of wild-type or revertant viruses at 15 days (Fig. 5B, left). At an MOI of 3, mutant virions were detected at 5 days after infection, but the maximum mutant virus titers were 500-fold less than those of wild-type and revertant viruses at 7 days post infection (Fig. 5B, right). This replication defect of IE1(Δ290–320) virus was 10-fold less in PML-knockdown (shPML) cells than in control (shC) cells, indicating that the PML targeting activity of IE1 indeed promoted viral replication (S4C Fig). Considering that IE1 exon-4-deleted mutant virus (CR208) grew as efficiently as wild-type virus at high MOIs in normal fibroblast [44], it is likely that MOI of 3 IFU per cell used in our analysis was not high enough for IE1(Δ290–320) virus to grow as efficiently as wild-type virus. When we compared the growth of IE1(Δ290–320) and CR208 viruses, both viruses displayed an MOI-dependent growth pattern but IE1(Δ290–320) virus grew slightly faster than CR208, suggesting that the activities of IE1 other than PML targeting may also contribute to viral replication (S4D Fig).

We also examined the accumulation of viral proteins after infection at an MOI of 3 (Fig. 5C). At 6 h after infection, the level of IE1(Δ290–320) in mutant virus-infected HF cells was comparable to intact IE1 levels in wild-type and revertant virus-infected cells. However, at 1 day after infection, the level of IE1(Δ290–320) was significantly lower in mutant virus-infected cells. Interestingly, over the first 24 h, the level of 86-kDa IE2 in mutant virus-infected cells increased to levels higher than those of wild-type and revertant virus-infected cells, but then decreased to lower levels. Consequently, the accumulation of an early protein p52 (encoded by UL44) was significantly impaired in mutant virus infection. These results show that IE1(Δ290–320) virus has a severe growth defect, which is due, at least in part, to the reduced accumulation of viral IE proteins. The level of IE1(Δ290–320) continued to increase until 15 h after infection (Fig. 5D), although IE1(Δ290–320) was less stable than wild-type IE1. Level of IE1(Δ290–320) was lost in cells treated with cycloheximide independent of proteasome activity (Fig. 5D).

To investigate the effect of IE1-PML interaction on type I IFN-induced ISG expression during virus infection, we compared ISG mRNA levels in HF cells infected with wild-type and mutant viruses. qRT-PCR assays were performed on cells infected with viruses at an MOI of 3 for 12 h. The results revealed that the transcription of ISG54 and CXCL10 was strongly induced by UV-HCMV or CR208, confirming a critical role of IE1 in the downregulation of ISG expression (Fig. 5E). However, these increases in ISG54 and CXCL10 mRNA levels were much lower for IE1(Δ290–320) and IE1(Δ421–475) virus infections (Fig. 5E). In a control experiment, INFβ mRNA levels were not different in cells infected with wild-type, UV-HCMV, or mutant virus (Fig. 5E). The numbers of IE1-positive cells and the expression levels of IE1 at the time points of qRT-PCR assays were comparable for cells infected with wild-type, IE1(Δ290–320), or IE1(Δ421–475) viruses as determined by IFA (Fig. 5F) and immunoblot analysis (Fig. 5G). The amounts of input viral DNAs were also comparable (Fig. 5F). These results demonstrate that a lack of PML binding in IE1(Δ290–320) virus infection leads to reduction of IE1 activity to downregulate ISG transcription.

We also investigated whether the reduced activity of IE1(Δ290–320) in suppressing ISG transcription is found in the context of the clinically related HCMV strain. We produced recombinant viruses expressing wild-type or Δ290–320 mutant IE1 in the context of the Toledo strain and compared their abilities to inhibit ISG54 and CXCL10 expression. The results showed that like the Towne-based mutant virus, the IE1(Δ290–320)-expressing Toledo virus less efficiently suppressed ISG54 and CXCL10 transcription compared to the wild-type virus, indicating that this IE1 activity is conserved in different HCMV strains (S5 Fig).

### IE1 forms a complex with STAT1, STAT2, HDAC1, HDAC2, and PML during infection

**Fig 6:**
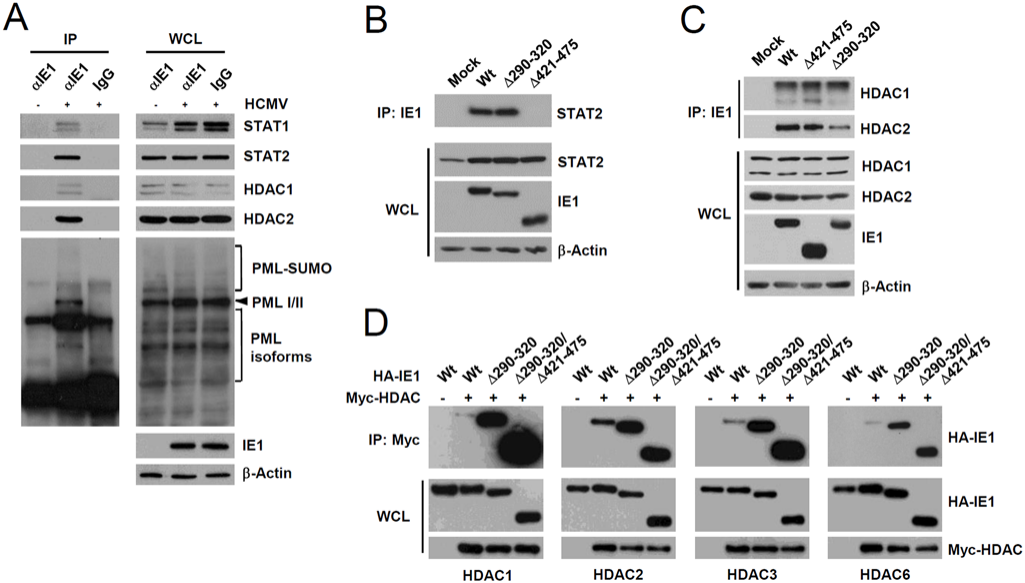
Association of IE1 with STAT1, STAT2, HDAC1, HDAC2, and PML during infection and the binding of IE1(Δ290–320) with STAT2 and HDACs. (A) HF cells were mock-infected (-) or infected (+) with HCMV (Towne) at an MOI of 2 IFU per cell. At 24 h after infection, total cell lysates were prepared and immunoprecipitated with anti-IE1 antibody (CH443); immunoprecipitation with mouse IgG was used as a negative control. The samples were subjected to SDS-PAGE and immunoblotted with antibodies for the indicated proteins (left panels). Immunoblot assays were also performed with total cell lysates to confirm protein expression levels (right panels). (B and C) HF cells were mock-infected or infected with wild-type, IE1(Δ290–320), or IE1(Δ421–475) mutant virus at an MOI of 2. At 12 h, total cell lysates were prepared and immunoprecipitated with anti-IE1 antibody (CH443) and immunoblotted with anti-STAT2 antibody (B) or with anti-HDAC1 or anti-HDAC2 antibodies (C). Total cell lysates were also immunoblotted with antibodies for IE1 (CH443), STAT2, HDAC1, and HDAC2 to confirm protein expression levels. (D) 293T cells were cotransfected with plasmids expressing HA-tagged wild-type, Δ290–320, or Δ290–320/Δ421–475 IE1 and myc-HDAC1, myc-HDAC2, myc-HDAC3, or myc-HDAC6 as indicated. At 48 h, whole cell lysates were prepared and immunoprecipitated with anti-Myc antibody and immunoblotted with anti-HA antibody. Levels of HA-IE1 and myc-HDAC proteins in whole cell lysates were determined by immunoblotting.

Since PML interacted with STAT1, STAT2, HDAC1, and HDAC2, we investigated whether IE1 simultaneously interacts with PML, STAT1, STAT2, and HDACs during infection. When HF cells were infected with HCMV for 24 h and co-IP assays were performed, IE1 was coimmunoprecipitated with STAT1, STAT2, HDAC1, HDAC2, and PML (Fig. 6A), whereas immunoprecipitation with control IgG did not coprecipitate any of these proteins in the control (Fig. 6A). Furthermore, the result of gel filtration analysis demonstrated the existence of the high molecular mass fractions (>400-kDa) containing IE1, PML, STAT2, HDAC2, and HDAC1, but not IRF9 and p52 (encoded by UL44) (S6 Fig). The colocalization of IE1 with HDAC1 and HDAC2 was also observed in HF cells during the early stage of virus infection (S7 Fig). These results indicate that IE1 may form a protein complex containing STAT1, STAT2, HDAC1, HDAC2, and PML during infection. Furthermore, IE1 interacted with all nuclear PML isoforms in cotransfection/co-IP assays (S8A Fig).

### A small deletion within the hydrophobic core of IE1 does not affect its interactions with STAT2 and HDACs

We also tested whether the reduced activity of IE1(Δ290–320) in ISG suppression is related with a reduction of its ability of binding STAT2 or HDACs. When HF cells were infected with wild-type, IE1(Δ290–320), or IE1(Δ421–475) viruses for 12 h and co-IP assays were performed, both wild-type IE1 and IE1(Δ290–320) were coprecipitated with comparable amounts of STAT2; however, IE1(Δ421–475) did not interact with STAT2 (Fig. 6B), thus demonstrating that IE1(Δ290–320) interacts with STAT2 as efficiently as wild-type IE1. Consistent with the notion that the central hydrophobic region of IE1 and its associated PML-binding activity are not required for STAT2 binding, IE1(L174P) mutant, which could not bind PML [28], still interacted with STAT2 in co-IP assays (S8B Fig). In similar co-IP assays performed using wild-type or IE1 mutant virus-infected cell lysates, IE1(Δ290–320) and IE1(Δ421–475) were coimmunoprecipitated with HDAC1 and HDAC2 like wild-type IE1 (Fig. 6C), indicating that IE1(Δ290–320) still associates with HDAC1 and HDAC2 in virus-infected cells. Consistently, IE1 was mapped to require the N-terminal regions for HDAC2 and HDAC1 binding in co-IP assays (S9 Fig). HDAC3 and HDAC6 have been previously shown to promote type I IFN signaling [45–47]. We also found that HDAC3 and HDAC6 interacted with wild-type IE1 and interacted even more strongly with Δ290–320 and Δ290–320/Δ421–475 mutant proteins (Fig. 6D). Taken together, these results show that reduced ISG suppression by IE1(Δ290–320) is not due to reduction in binding to STAT2 or HDACs, demonstrating the importance of IE1-PML interaction for the inhibition of ISG transcription.

### IE1 inhibits the association of PML, STAT2, and HDAC1 with ISG promoters during virus infection

**Fig 7:**
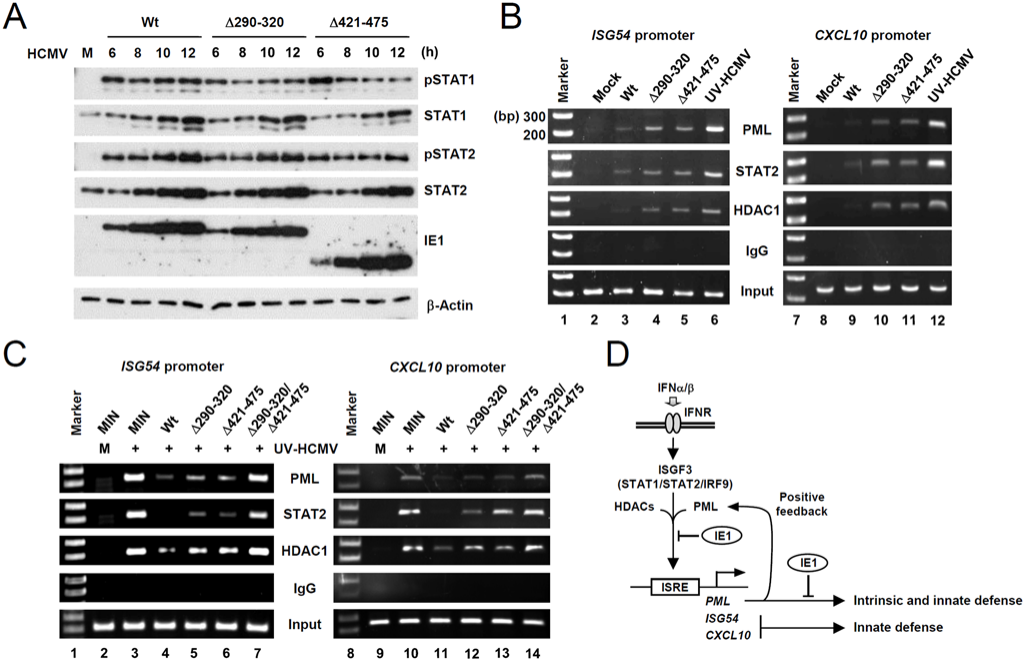
Comparison of the levels of the total and activated forms of STAT1 and STAT2, and the association of PML, STAT2, and HDAC1 with ISG promoters during wild-type or IE1 mutant virus infection. (A) HF cells were infected with wild-type, IE1(Δ290–320), or IE1(Δ421–475) virus, at an MOI of 3 IFU per cell for the indicated times. Total cell lysates were prepared immunoblotted with antibodies for STAT1, STAT2, their phosphorylated forms, and IE1 (6E1). (B) HF cells were mock-infected or infected with wild-type, IE1(Δ290–320), IE1(Δ421–475) virus or UV-HCMV at an MOI of 3 for 12 h. ChIP assays were performed with anti-PML (PG-M3), anti-STAT2, anti-HDAC1 antibodies or with control IgG to detect the amounts of these proteins bound to ISG54 and CXCL10 promoters. The sizes of DNA fragments amplified from ISG54 and CXCL10 promoters were 199 bp and 241 bp, respectively. The 100 bp DNA ladder size markers are shown. (C) Control (MIN) and IE1 (wild-type or mutant)-expressing HF cells were mock-infected or infected with UV-HCMV at an MOI of 3 for 24 h and ChIP assays were performed as described in (B). (D) Model for the presence of a positive feedback loop for PML expression, the roles of PML in intrinsic defense and type I IFN signaling, and their regulation by HCMV IE1.

To further investigate the mechanisms by which the binding of PML by IE1 inhibits type I IFN-induced ISG transcription, we compared STAT1 and STAT2 levels in wild-type, IE1(Δ290–320), and IE1(Δ421–475) virus-infected HF cells. The results showed that levels of STAT1 and STAT2 and of their activated forms were comparable in cells infected with wild-type or mutant viruses (Fig. 7A). These results suggested the IE1-PML interaction did not significantly affect STAT1 or STAT2 levels.

We next compared the association of PML, STAT2, and HDAC1 with ISG54 and CXCL10 promoters in wild-type and mutant virus infected cells using ChIP assays. The results revealed that wild-type virus infection more substantially reduced the association of PML, STAT2, and HDAC1 with both ISG promoters than UV-HCMV infection (Fig. 7B, compare lanes 3 and 6; 9 and 12). However, Δ290–320 and Δ421–475 mutant viruses less effectively reduced those associations (Fig. 7B, lanes 4 and 5; 10 and 11). These results demonstrate that, the lack of PML binding in IE1(Δ290–320) mutant virus infection resulted in less effective sequestration of PML, STAT2, and HDAC1 from ISG promoters. When we compared the ChIP analysis after infection with IE1(Δ290–320) and CR208 viruses, the results with IE1(Δ290–320) virus were similar to those with CR208 virus (S10 Fig).

Similar ChIP assays were performed on wild-type or mutant IE1-expressing HF cells infected with UV-HCMV. The results showed that UV-HCMV infection resulted in high level associations of PML, STAT2, and HDAC1 with both ISG54 and CXCL10 promoters compared to mock-infection in control cells (Fig. 7C, compare lanes 2 and 3; 9 and 10). While the expression of wild-type IE1 substantially reduced these associations (Fig. 7C, lanes 4 and 10), IE1(Δ290–320) and IE1(Δ421–475) reduced these associations less effectively (Fig. 7C, lanes 5 and 6; 12 and 13). IE1(Δ290–320/Δ421–475) almost completely lost this repressive activity (Fig. 7C, lanes 7 and 14). These results demonstrate that both PML binding and STAT2 binding by IE1 contribute to the ability of IE1 in inhibiting the associations of PML, STAT2, and HDAC1 with ISG promoters.

## Discussion

Accumulating evidence indicates that in addition to acting as a nuclear restriction factor for incoming viral genomes in intrinsic defense, PML also plays a critical role in innate immunity. Initial findings regarding the positive role of PML in IFNγ-induced MHC class I gene expression were controversial [48, 49], but recent studies have demonstrated that PML positively regulates IFNγ signaling by upregulating STAT1 phosphorylation [38] and that PML promotes IFNγ-induced MHC class II gene expression by stabilizing class II transactivator [39]. The present study shows that PML also acts as a positive regulator of type I IFN-induced ISG expression. In IFNβ-treated or UV-HCMV-infected cells, PML was required for the efficient accumulation of the activated forms of STAT1 and STAT2. Furthermore, after UV-HCMV infection, PML formed a protein complex with STAT1, STAT2, HDAC1, and HDAC2 and associated with ISG promoters. Therefore, PML appears to contribute to type I IFN-induced ISG transcription by regulating the expression of STAT1 and STAT2 and directly acting on ISG promoter to regulate ISGF3. The results from our study, together with those previously reported, indicate a general role of PML in both type I and type II IFN responses. Moreover, because PML is induced by IFNs, these PML activities demonstrate a positive feedback loop for IFN signaling (Fig. 7D).

We found that PML could promote STAT1 transcription in unstimulated HF cells. The mechanism underlying this PML activity is not clear. STAT1 promoter is IFN-inducible, and therefore, the reduced transcription of STAT1 in PML-knockdown cells may simply reflect the lack of positive ISGF3 regulation due to the absence of PML activity. However, our analysis of cells treated with anti-IFNAR antibody suggested that PML also directly activates STAT1 expression. STAT1 promoter also contains repressor element-1 (RE-1) that is recognized by the RE-1 silencing transcription factor (REST) [50], which recruits CoREST, HDACs, and histone demethylase to suppress STAT1 gene transcription [51, 52]. However, it remains to be determined whether PML depletion affects the activity of CoREST/REST complex. We also found that PML depletion rendered STAT2 more vulnerable to proteasomal degradation. Although no ubiquitin E3 ligase has been identified for STAT2 degradation under normal cell conditions, PML might sequester E3 ligases in the nucleus to protect STAT2 from proteasomal degradation.

We showed that PML was associated with STAT1, STAT2, HDAC1, and HDAC2, and with ISG promoters, which concurs with previous findings regarding the direct interactions between PML and HDAC1 and HDAC2 [43] and the association between PML and STAT1 in IFNγ-treated cells [53]. Furthermore, we found that during HCMV infection IE1 reduced the association of PML, STAT2, and HDAC1 with ISG promoters. Both binding to STAT2 and binding to PML by IE1 independently contributed to ISG downregulation, but binding to HDACs did not. The potential regulation of type I IFN response by the interaction between IE1 and HDACs was not addressed in the present study. Nevertheless, our data demonstrate that the reduced activities of Δ290–320 and Δ290–320/Δ421–475 IE1 mutants with respect to the repression of ISG transcription are not due to a loss in their abilities to interact with HDAC1, HDAC2, HDAC3, and HDAC6. Notably, HDAC1 and HDAC2 have been shown to participate in the upregulating IRF3 phosphorylation following γ-herpesvirus infection [54]. However, PML does not seem to control this HDAC activity during HCMV infection, since PML knockdown did not significantly affect IRF3 phosphorylation after UV-HCMV infection. Furthermore, the binding to HDACs by IE1 does not appear to affect IFNβ production in HCMV infection, because the levels of IFNβ mRNA in wild-type and IE1(Δ290–320) mutant virus-infected cells were comparable.

Recently, it was demonstrated that the specific PML isoform, PML-IV, participates in the production of IFNβ. More specifically, the overexpression of PML-VI augmented the production of IFNβ in U373-MG or HeLa cells stimulated by double RNA or virus infection, and this augmentation was attributed to the ability of PML-VI to recruit Pin1 to PML NBs, and thereby prevent the degradation of phosphorylated IRF3 [40]. We also observed that the overexpression of PML-IV in normal HF cells slightly increased IRF3 activation after vesicular stomatitis virus infection, although this was not observed after HCMV infection. Moreover, depletion of endogenous PML in HF cells by shRNA did not apparently affect UV-HCMV-induced IRF3 activation. These different results could stem from the use of different cell types or viruses. Nevertheless, our results provide evidence that PML facilitates ISG transcription after IFNβ production by regulating the accumulation of activated STAT1 and STAT2 and by interacting with them and with HDACs on ISG promoters. Accordingly, PML appears to positively regulate type I IFN signaling at multiple steps before and after IFN production.

During herpesvirus infection, PML plays an essential role in intrinsic suppression of the viral genome. HCMV IE1 is responsible for overcoming this PML-mediated intrinsic suppression. Considering a new role of PML in type I and type II IFN signaling, it appears that the IE1-PML interaction represents a critical viral strategy to evade both intrinsic defense and innate immunity. These functions of PML in host antiviral defense may explain why PML targeting activity is widely conserved among many DNA viruses.

## Materials and Methods

### Cell culture and viruses

Human dermal fibroblast (HF) (ATCC) and human embryonic kidney (HEK) 293 and 293T cells (ATCC) were grown in Dulbecco's modified Eagle's medium (DMEM) supplemented with 10% fetal bovine serum and 100 units/ml of penicillin and 100 μg/ml of streptomycin. The HCMV Towne virus stocks were prepared as previously described [28]. For comparative analysis, the stocks of wild-type virus, IE1-deledeted CR208 virus, and other IE1 mutant viruses encoding IE1(Δ290–032) or IE1(Δ421–475) were prepared in IE1-expressing HF cells and the titers of virus stocks were determined as infectious units (IFU) after performing infectious center assays in normal HF cells (see below). To produce UV-inactivated HCMV (UV-HCMV), the virus stock was irradiated with UV light three times at 0.72 J/cm^2^ using a CL-1000 cross-linker (UVP).

### Infectious center assays

To perform virus infectivity assays, the diluted samples were used to inoculate a monolayer of HF cells (1 x 10^5^) in a 24-well plate. At 24 h post-infection, cells were fixed with 500 μl of cold methanol for 10 min. Cells were then washed three times in phosphate-buffered saline (PBS), incubated with anti-IE1 rabbit polyclonal antibody (PAb) in PBS at 37°C for 1 h, followed by incubation with phosphatase-conjugated anti-rabbit immunoglobulin G (IgG) antibody in PBS at 37°C for 1 h. Finally, the cells were gently washed in PBS and treated with 200 μl of nitroblue tetrazolium/5-bromo-4-chloro-3-indolylphosphate (KPL) at room temperature for 1 h, according to the manufacturer's instructions. The IE1-positive cells were counted in at least three to five separate fields per well under a light microscope (200X magnification).

### Plasmids

The mammalian expression plasmids for N-terminal HA-tagged or untagged IE1 (wild-type or mutant) were described previously [28, 33]. The wild-type and mutant IE1 DNAs (Towne strain) were cloned into pENTR vectors (Invitrogen). Retroviral vectors encoding wild-type or mutant IE1 were produced by transferring the IE1 DNAs from pENTR vectors to pMIN [55]-based destination vector using LR Clonase (Invitrogen). The PML, HDAC1, HDAC3, and HDAC6 cDNAs were PCR cloned into pENTR vectors. Plasmids expressing myc-tagged PML or HDACs were produced by transferring the DNAs to pCS3-MT (with a 6Myc tag) [56]-based destination vector using LR Clonase. The plasmid expressing myc-HDAC2 was described previously [57]. pSIREN-RetroQ (BD Biosciences) retroviral vectors encoding short hairpin RNA (shRNA) for PML (shPML) or control shRNA (shC) [29] were as previously described [58].

### Electroporation and DNA transfection

Bacmid DNAs were introduced into HF cells by electroporation. For each reaction, HF cells (2 x 10^6^) in 400 μl of resuspension buffer were mixed with 5 μg of Towne (T)-BAC DNA, 4.5 μg of plasmid pCMV71 encoding transactivator pp71, and 0.5 μg of pEGFP-C1 (to monitor electroporation efficiency). After electroporation at 1,300 V and 40 ms using a Microporator MP-100 (Digital Bio Technology), cells were plated into T-25 flasks. When surviving cells became confluent, cells were split into new flasks at a ratio of 1:2.

For luciferase reporter assays and co-immunoprecipitation (co-IP) assays, 293T cells were transfected using the *N*,*N*-bis-(2-hydroxyethyl)-2-aminoethanesulfonic acid-buffered saline (BBS; Calbiochem) version of the calcium phosphate method. Briefly, a mixture of plasmid and sterile H_2_O was mixed with CaCl_2_ (to a final concentration of 0.25 M) and with an equal volume of 2X BBS (50 mM BBS [pH 7.0], 280 mM NaCl, 1.5 mM Na_2_HPO_4_). This mixture was kept at room temperature for 20 min and added drop wise to cells.

### siRNA transfection

For PML knockdown using siRNA, On-TARGET plus SMARTpool siRNAs (Dharmacon) specifically targeting PML (catalog no. J-006547-07) [59] and non-targeting control pools of siRNAs (catalog no. D-001210-01) were used. The transfection of HF cells with siRNA was performed using 20 nM of siRNA using Dharmafect (Dharmacon) according to the manufacturer’s instructions.

### Luciferase reporter assay

Cells were lysed using three freeze-thaw cycles in 100 μl of 0.25 M Tris-HCl (pH 7.9) containing 1 mM dithiothreitol. Subsequent procedures were performed as described previously [60]. A TD-20/20 luminometer (Turner Designs) was used for the 10-s assay to measure photons produced.

### Antibodies

Mouse monoclonal antibody (MAb) 810R, which can detect both IE1 and IE2 proteins, and mouse MAb 6E1specific for IE1 were purchased from Chemicon and Vancouver Biotech, respectively. Mouse MAbs against IE1 (CH443), ICP36 (p52; UL44) and pp28 (UL99) were purchased from Virusys. Mouse MAb anti-β-actin and rat MAb anti-HA (3F10) conjugated with peroxidase were purchased from Sigma and Roche, respectively. Mouse MAbs for PML (PG-M13) and PKR and rabbit PAbs for STAT1 (E-23), STAT2 (C-20), IRF3 (FL425), and IRF9 (H-143) were purchased from Santa Cruz. Rabbit PAbs for p-STAT1 (Tyr701) and p-IRF3 (Ser396) were purchased from Cell Signaling. Rabbit PAb for p-STAT2 (Tyr689) and mouse MAb for HDAC1 were purchased from Upstate. Rabbit PAb for HDAC2 was obtained from Zymed. Mouse MAb for human IFNα/β receptor chain 2 (MAB1155) were purchased from Millipore. Anti-peptide rabbit PAb PML(C) specific for PML was described previously [23]. Mouse MAb anti-PML (5E10) [61] was kindly provided by Gary S Hayward (Johns Hopkins University School of Medicine, Baltimore, USA).

### Indirect immunofluorescence assay (IFA)

Cells were washed in PBS, fixed in cold methanol at 4°C for 5 min, and then permeabilized with cold PBS buffer for 5 min. Cells were incubated with appropriate primary antibodies in PBS at 37°C for 1 h and then with affinity-purified fluorescein isothiocyanate (FITC)-labeled or rhodamine/redX-coupled immunoglobulin G (IgG) (Jackson ImmunoResearch Lab.) at 37°C for 1 h. Slides were examined and photographed using a Carl Zeiss Axiophot microscope. For confocal microscopy, a Carl Zeiss Axioplan 2 confocal microscope system running LSM510 software (Carl Zeiss) was used.

### Immunoblot analysis

Cells were washed with cold PBS and then placed in 1X sample buffer. Cell lysates were prepared by boiling, separated by SDS-PAGE, and then transferred onto nitrocellulose membranes (GE Healthcare). Membranes were blocked for at least 1 h in PBST (PBS plus 0.1% Tween 20 [Sigma]) containing 5% skim milk and then washed with PBST. After incubation with appropriate antibodies, proteins were visualized using an enhanced chemiluminescence system (Roche).

### Retrovirus transduction and the selection of stably transduced cells

292T cells were cotransfected with pSIREN-RetroQ plasmids expressing shC or shPML and packaging plasmids pHIT60 (expressing murine leukemia virus Gal-Pol) and pMD-G (expressing the envelope G protein of vesicular stomatitis virus) [29] using Metafectene reagents (Biotex). Cell supernatants harvested at 48 h after transfection were used to transduce HF cells in the presence 7.5 μg per ml of polybrene (Sigma). Cells were selected with 2 μg per ml of puromycin (Calbiochem). Selected cells were maintained in medium containing 0.5 μg per ml of puromycin. To generate IE1 or PML-expressing HF cells, recombinant pMIN-based retroviruses were prepared as described above. HF cells were transduced with retroviruses in a medium supplemented with 0.4 mg per ml of G418 (Calbiochem) to select stably transduced cells. The selected cells were then maintained in a medium containing 0.1 mg per ml of G418.

### RNA isolation and quantitative real-time reverse transcription-PCR (qRT-PCR)

Total RNAs were isolated from 2 x10^5^ cells using TRI reagent (Molecular Research Center, Inc) and a MaXtract High Density tube (QIAGEN). cDNAs were synthesized using random hexamer/oligo d(T) primers provided by the QuantiTect Reverse Transcriptase Kit (QIAGEN). Quantitative real-time RT-PCR was performed using SYBR green PCR core reagents (Applied Biosystems) and ABI Prism SDS software. PCR was performed using **t**he following primers: 5′-GACATCCCTGAGATTAAG-3′ (for IFNβ forward), 5′-ATGTTCCTGGAGCATCTCG-3′ (for IFNβ reverse), 5′-GCTTAGACATATTCTGAGCCTAC-3′ (for CXCL10 forward), 5′-AGCTGATTTCCTGACCATCATTG-3′ (for CXCL10 reverse), 5′-AGAAATCAAGGGAGAAAGAA-3′ (for ISG54 forward), 5′-AAGGTGACTAAGCAAATGGT-3′ (for ISG54 reverse), 5′-AGCGGGAAATCGTGCGTG-3′ (for β-actin forward), 5′-CAGGGTACATGGTGGTGCC-3′ (for β-actin reverse), 5’-ACTTTTTCCTGGCTCATCTC-3’ (for PKR forward), 5’-ACATGCCTGTAATCCAGCTA-3’ (for PKR reverse), 5’-GCTGGGACCTGACGGTG-3’ (for ISG15 forward), 5’-TTAGCTCCGCCCGCCAG-3’ (for ISG15 reverse), 5’-CAGAAAGCAGTGCGGCCCCT-3’ (for USP18 forward), 5’-TGCAGGGCGTCCTCCAGTGT-3’ (for USP18 reverse), 5’-CACACCAGCGTTTTGACCAG-3’ (for ChREBP forward), 5’-ACTCAAACAGAGGCCGGATG-3’ (for ChREBP reverse), 5’-CAACCTGCTGAAGGAGAAG-3’ (for c-Fos forward), and 5’-AGATCAAGGGAAGCCACAGAC-3’ (for c-Fos reverse).

### qPCR for input viral DNA

Virus-infected cells (2 x10^5^ cells) were trypsinized and collected by centrifugation. Total DNAs were isolated from cells using the QIAamp DNA Mini Kit (QIAGEN). Quantitative real-time PCR (qPCR) to measure the amounts of input viral DNA was performed using SYBR green PCR core reagents (Applied Biosystems) and ABI Prism SDS software. The PCR primers to amplify the IE1 exon 4 were used: 5′-ATAAGCGGGAGATGTGGATG-3′ (forward) and 5′-TTCATCCTTTTTAGCACGGG-3′ (reverse).

### Co-IP assays

Cells were harvested and sonicated in 1 ml of CoIP buffer (50 mM Tris-Cl [pH 7.4], 50 mM NaF, 50 mM sodium pyrophosphate, 0.1% Triton X-100, and protease inhibitor cocktail [Sigma]) using a Sonic Dismembrator (Model 500, Fisher Scientific) for 15 s (pulse on 1 s; pulse off 1 s). Cell lysates were incubated with appropriate antibodies for 16 h at 4°C, 30 μl of a 50% slurry of protein G-Sepharose (Millipore) was added. The mixture was incubated for 2 h at 4°C and centrifugated. The pellet was washed at least seven times with Co-IP buffer without protease inhibitor cocktail. The beads were suspended and boiled for 7 min in 1X sample buffer. Each sample was subjected to SDS-PAGE. Immunoblot assays were performed with appropriate antibodies. To remove DNA and RNA in cell lysates, 100 U/ml of DNase (Roche) and 10mg/ml of RNase (Sigma) were used to treat cell lysates for 12 h at 4°C prior to immunoprecipitation.

### Chromatin immunoprecipitation (ChIP) assay

ChIP assays were performed using a kit according to the manufacturer's instructions (Upstate Biotechnology) with minor modifications. Briefly, UV-HCMV-infected control or PML-knockdown HF cells (1 x 10^6^) were fixed with 1% formaldehyde for 10 min at 8 h after infection. Cells were lysed with SDS-lysis buffer. ChIP assays were performed using appropriate antibodies or control IgG. One percent of the lysate was reserved to allow quantitation of the DNA present in samples prior to immunoprecipitation. To detect human ISG54 and CXCL10 promoter DNAs, DNAs purified from immunocomplexes were amplified by PCR using the following primers: 5′-GGAGGAAAAAGAGTCCTCTA-3 (for ISG54P forward) and 5-AGCTGCACTCTTCAGAAA-3′ (for ISG54P reverse) to amplify the ISG54 promoter region from -169 to +30, and 5′-TTTGGAAAGTGAAACCTAATTCA-3 (for CXCL10P forward) and 5-AATTGAGGAATGTCTCAGAAAA-3′ (for CXCL10P reverse) to amplify the CXCL10 promoter region from -224 to +17. A portion (5 μl) of the total 50 μl of precipitated DNA samples was used for PCR. The PCR program consisted of 95°C for 10 min, followed by 35 amplification cycles (94°C for 30 s, 48°C for 1 min, and 68°C for 30 s) for ISG54 or 30 amplification cycles (94°C for 30 s, 53°C for 1 min, and 68°C for 30 s) for CXCL10, and a final extension step of 68°C for 10 min.

### Statistical analysis

Statistical significances were determined using the Student’s *t*-test and are indicated by *P<0.05, **P<0.01, or ***P<0.001.

## Supporting Information

S1 Fig PML-knockdown by shRNA-expressing retroviral vector transduction and siRNA transfection in HF cells and re-expression of PML-I. (A and B) Control and PML-knockdown HF cells were produced using the retroviral vectors (Puro^+^) expressing control (shC) or PML-specific shRNA (shPML). Expression levels of PML were determined by immunoblotting (with anti-PML antibody 5E10) (A) and by IFA [using anti-PML antibody PML(C)] (B). DAPI was used to stain the nuclei. (C) HF cells were transfected with 20 nmole of control scrambled (siC) or PML-specific siRNA (siPML) twice at times 0 and 24 h. At 72 h, cells were mock-infected or infected with UV-HCMV at an MOI of 5 for 6 and 8 h. PML-knockdown was determined by immunoblotting with PML(C) antibody. Open circles indicate non-specific bands. (D) shC and shPML HF cells were transduced using empty retroviral vectors (EV) or PML-I-expressing vectors. The re-expression of PML-I in shPML cells was determined by immunoblotting with PML(C) antibody. Open circles indicate non-specific bands. (E) shPML HF cells were cotransfected with 0.5 μg of the ISG54 ISRE-Luc reporter plasmid and 1 μg of empty vector or plasmid encoding myc-PML-I as indicated. At 24 h, cells were untreated or treated with IFNβ (1 x 10^3^ units/ml) for 8 h, and luciferase reporter assays were performed. Expression levels of PML-I were determined by immunoblotting with anti-myc antibody.
(TIF)

S2 Fig Effects of PML knockdown on IFNβ-mediated ISG induction and the transcription of STAT1 and STAT2 in 293 cells. (A) Control (shC) and PML-knockdown (shPML) 293 cells produced using retroviral vectors were untreated or treated with IFNβ (1 x 10^3^ units/ ml) and the mRNA levels of ISG54, CXCL10, and PKR were measured by qRT-PCR. (B) The mRNA levels of STAT1 and STAT2 in control (shC) and PML-knockdown (shPML) 293 cells were measured by qRT-PCR.
(TIF)

S3 Fig Association of PML with STAT1, STAT2, and HDAC1 on ISG54 and CXCL10 promoters after IFNβ treatment. (A) Normal HF cells were treated or not with IFNβ (1 x 10^3^ units/ ml) for 8 h and co-IP assays were carried out. Total cell lysates were prepared and immunoprecipitated with anti-PML antibody (PG-M3) or mouse IgG as a negative control. Immunoprecipitated samples and whole cell lysates were subjected to SDS-PAGE and then immunoblotted with antibodies for STAT1, STAT2, HDAC1, HDAC2, IRF9, ribonucleotide reductase R1, and PML (PG-M3). Circles indicate non-specific bands. (B) HF cells were treated or not with IFNβ as described in (A) and ChIP assays were performed using anti-PML (PG-M3), anti-STAT2, anti-HDAC1, and anti-HDAC2 antibodies. PCR was performed to detect ISG54 and CXCL10 promoter DNAs. The sizes of the DNA fragments amplified from the ISG54 and CXCL10 promoter regions were 199 bp and 241 bp, respectively. A 100 bp DNA ladder was used as a size marker.
(TIF)

S4 Fig Analyses of IE1-expressing HF cells and IE1(Δ290–320) mutant virus. (A) Normal HF and IE1-expressing HF (HF-IE1) cells were mock-infected or infected with CR208 at an MOI of 1 IFU per ml. The phase contrast images were taken at 6 days after infection. The CPE was evident in HF-IE1 cells but not in HF cells after CR208 infection, demonstrating that HF-IE1 cells effectively support the growth of CR208. (B) HF cells were mock-infected were infected with wild-type or IE1(Δ290–320) virus. At 6 h after infection, cells were fixed in methanol and double-label IFA was performed with anti-IE1 (6E1) and anti-PML (PML-C) antibodies. The images were obtained with a Carl Zeiss Axioplan 2 confocal microscope system. (C) shC and shPML HF cells were infected with wild-type or IE1(Δ290–320) mutant virus at an MOI of 3 IFU per cell. At 6 days after infection, the total numbers of infectious units in culture supernatants were determined using infectious center assays. (D) HF cells were infected with wild-type, IE1(Δ290–320) mutant, or CR208 virus at an MOI of 1, 3, or 5 IFU per cell. At 5 days after infection, the total numbers of infectious units in culture supernatants were determined as in (C).
(TIF)

S5 Fig Production and analysis of the Toledo virus expressing IE1(Δ290–320). (A) The scheme of the production of a recombinant HCMV (Toledo) virus encoding IE1(Δ290–320). The Toledo-BAC clone was a gift from Hua Zhu (UMDNJ-New Jersey Medical School, Newark, NJ, USA). The Toledo-BAC clone encoding IE1(Δ290–320) protein was produced by using a counter-selection BAC modification kit (Gene Bridges). Briefly, the rpsL-neo cassette DNA was PCR-amplified using LMV1912/1913 primers (see below) containing homology arms consisting of 50 nucleotides upstream and downstream of the target region plus 24 nucleotides homologous to the rpsL-neo cassette. The amplified rpsL-neo fragments with homology arms were purified and introduced into *E*. *coli* GS243 containing wild-type Toledo-BAC for recombination by electroporation using a Gene Pulser II (Bio-Rad). The intermediate Toledo-BAC constructs containing the rpsL-neo cassette were selected on Luria Broth (LB) plates containing kanamycin. Next, the rpsL-neo cassette was replaced by annealed oligo DNAs (LMV1914/1915) consisting of only homology arms (50 nucleotides upstream and downstream of the target region). The IE1(Δ290–320) Toledo-BAC was selected on LB plates containing streptomycin. LMV1912; 5’-ATATCCTCACTACATGTGTGGAGACCATGTGCAGTGAGTACAAGGTCACCGGCCTGGTGATGATGGCGGGATCG-3’, LMV1913; 5’-TTGATAACCTCAGGCTTGGTTATCAGAGGCCGCTTGGCCAGCAACACACTTCAGAAGAACTCGTCAAGAAGGCG-3’, LMV1914; 5’-ATATCCTCACTACATGTGTGGAGACCATGTGCAGTGAGTACAAGGTCACCAGTGTGTTGCTGGCCAAGCGGCCTCTGATAACCAAGCCTGAGGTTATCAA-3’, and LMV1915; 5’-TTGATAACCTCAGGCTTGGTTATCAGAGGCCGCTTGGCCAGCAACACACTGGTGACCTTGTACTCACTGCACATGGTCTCCACACATGTAGTGAGGATAT-3’. (B) The wild-type and IE1(Δ290–320) Toledo-BAC clones were digested with SpeI and the pulse-field gel electrophoresis patterns of DNA fragments were shown. The arrowheads indicate the 12 kb and 2.7 kb DNA fragments in the wild-type BAC clone, which disappeared in the IE1(Δ290–320) BAC clone. (C) Recombinant Toledo viruses encoding wild-type IE1 and Δ290–320 mutant were grown in IE1(Towne)-expressing HF cells that received the Toledo-BAC (Wt) and Toeldo-BAC-IE1(Δ290–320) DNAs. HF cells were mock-infected or infected with wild-type and IE1(Δ290–320) viruses or UV-HCMV at an MOI of 2 IFU per cell. At 12 h post-infection, total RNAs were prepared and ISG54 or CXCL10 mRNA levels were determined by qRT-PCR. The results shown are the mean values and standard errors of three independent experiments. The comparable expression of IE1 in recombinant virus-infected cells at 12 h after infection was shown by immunoblotting using anti-IE1 (6E1) antibody.
(TIF)

S6 Fig IE1 forms a high molecular mass complex during HCMV infection. HF cells were infected with HCMV at an MOI of 3 IFU per cell for 24 h. The cell extracts (total 2 mg) were prepared and loaded onto a Superose6 10/300 GL column (GE Healthcare) pre-equilibrated with co-IP buffer. The proteins were eluted at 0.5 ml/min. Each fraction (15 μl) was analyzed by immunoblotting with antibodies for IE1, PML, STAT1, STAT2, HDAC, HDAC2, IRF9 and p52 (encoded by UL44). Apparent molecular mass was evaluated after column calibration with standard proteins [thyroglobulin (669-kDa), ferritin (440-kDa), aldolase (158-kDa), conalbumin (75-kDa), and ovalumin (44-kDa)] in the Gel Filtration Calibration Kit (GE Healthcare). The elution positions of these proteins are indicated at the top. The high molecular mass fractions (>400-kDa), which include IE1, PML, STAT2, HDAC2, and HDAC1, are indicated as dashed lines.
(TIF)

S7 Fig Colocalization of IE1 with HDAC2 and HDAC1 in virus-infected cells. HF cells were infected with HCMV (Towne) at an MOI of 1. Cells were fixed at 2 h (a-c and g-i) or at 6 h (d-f and j-o) after infection in methanol, and confocal double-label IFA was carried out for IE1 and HDAC2 (a-f), for IE1 and HDAC1 (g-l), or for IE2 and HDAC1 as a control (m-o).
(TIF)

S8 Fig Interactions of IE1 with PML isoforms and STAT2. (A) 293T cells were cotransfected with plasmids expressing HA-IE1 and myc-PML isoforms as indicated. At 48 h, total cell lysates were prepared and immunoprecipitated with anti-myc antibody and then immunoblotted with anti-HA antibody. Whole cells lysates were also immunoblotted with anti-HA or anti-myc antibodies. (B) Effect of Leu 174 to Pro substitution within the central hydrophobic region of IE1 on its interaction with STAT2. (Top) 293T cells were cotransfected with plasmids encoding HA-IE1 or HA-IE1(L174P) and myc-STAT2 as indicated. At 48 h, total cell lysates were prepared and immunoprecipitated with anti-myc antibody and then immunoblotted using anti-HA antibody. Whole cell lysates were also immunoblotted with anti-HA or anti-myc antibodies. (Bottom) Bar graph showing the relative amounts of wild-type or L174P mutant IE1 protein bound to myc-STAT2.
(TIF)

S9 Fig The interaction of IE1 with HDAC2 and HDAC1. (A) 293T cells were cotransfected with plasmids encoding myc-HDAC2 and untagged wild-type or mutant IE1 proteins (left) or HA-tagged IE1 proteins (right). At 48 h, Co-IP assays were performed using anti-myc antibody and this was followed by immunoblotting with anti-IE1 (6E1) (left) or anti-HA (right) antibodies. The expression levels of IE1 and HDAC2 in whole cell lysates were determined by immunoblotting. (B) Cells were cotransfected with plasmids encoding myc-HDAC1 and untagged or HA-tagged IE1 (wild-type or mutant) proteins as indicated. The Co-IP assay was conducted as described in (A). (C) Cells were cotransfected with plasmids encoding myc-tagged IE1 (wild-type or N-terminal truncated mutants) and HA-tagged HDAC1 or HDAC2 or UL44 (as a control) as indicated. The Co-IP assay was conducted as described in (A). (D) Interactions of wild-type and mutant IE1 proteins with HDAC1, HDAC2, STAT2, and PML are summarized as + (positive interaction) or—(negative interaction). nt: not tested; NLS: nuclear localization signal. The IE1 regions responsible for interactions with HDAC1, HDAC2, PML, and STAT2 are indicated.
(TIF)

S10 Fig Comparison of the association of PML, STAT2, and HDAC1 with ISG promoters during wild-type, IE1(Δ290–320) mutant, or CR208 virus infection. HF cells were mock-infected or infected with wild-type, IE1(Δ290–320), or CR208 virus at an MOI of 3 IFU per cell for 12 h. ChIP assays were performed with anti-PML (PG-M3), anti-STAT2, anti-HDAC1 antibodies or with control IgG to detect the amounts of these proteins bound to ISG54 and CXCL10 promoters. The sizes of DNA fragments amplified from ISG54 and CXCL10 promoters were 199 bp and 241 bp, respectively. The 100 bp DNA ladder size markers are shown.
(TIF)
