# Supplementary figures and images for: Positive Role of Promyelocytic Leukemia Protein in Type I Interferon Response and Its Regulation by Human Cytomegalovirus
Source: PLoS Pathog. 2015 Mar 26;11(3):e1004785. doi: 10.1371/journal.ppat.1004785 (PMC4374831; doi:10.1371/journal.ppat.1004785)

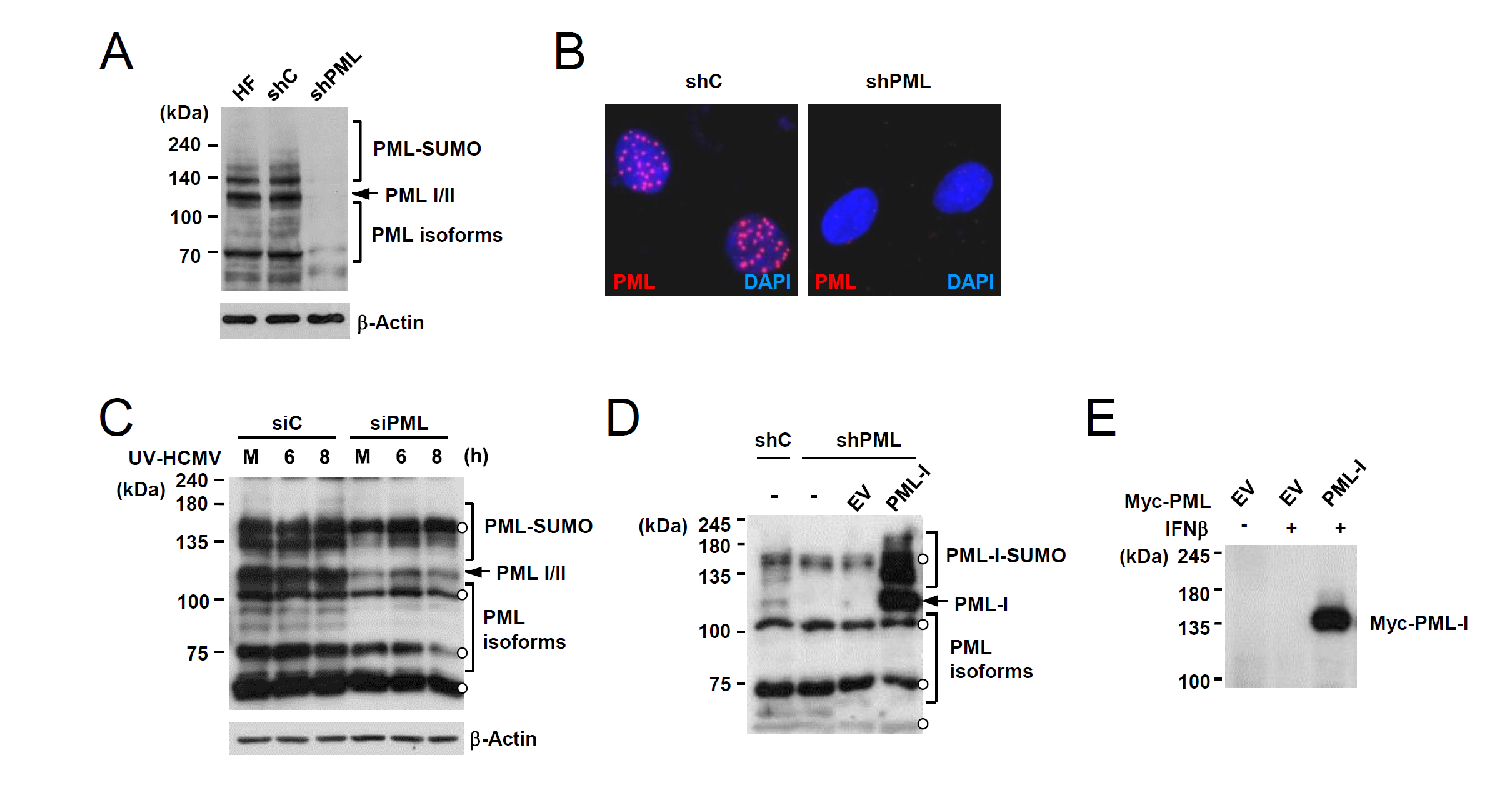

Supplement: S1 Fig — (A and B) Control and PML-knockdown HF cells were produced using the retroviral vectors (Puro+) expressing control (shC) or PML-specific shRNA (shPML). Expression levels of PML were determined by immunoblotting (with anti-PML antibody 5E10) (A) and by IFA [using anti-PML antibody PML(C)] (B). DAPI was used to stain the nuclei. (C) HF cells were transfected with 20 nmole of control scrambled (siC) or PML-specific siRNA (siPML) twice at times 0 and 24 h. At 72 h, cells were mock-infected or infected with UV-HCMV at an MOI of 5 for 6 and 8 h. PML-knockdown was determined by immunoblotting with PML(C) antibody. Open circles indicate non-specific bands. (D) shC and shPML HF cells were transduced using empty retroviral vectors (EV) or PML-I-expressing vectors. The re-expression of PML-I in shPML cells was determined by immunoblotting with PML(C) antibody. Open circles indicate non-specific bands. (E) shPML HF cells were cotransfected with 0.5 μg of the ISG54 ISRE-Luc reporter plasmid and 1 μg of empty vector or plasmid encoding myc-PML-I as indicated. At 24 h, cells were untreated or treated with IFNβ (1 x 10^3^ units/ml) for 8 h, and luciferase reporter assays were performed. Expression levels of PML-I were determined by immunoblotting with anti-myc antibody. (TIF) [file ppat.1004785.s001.tif]

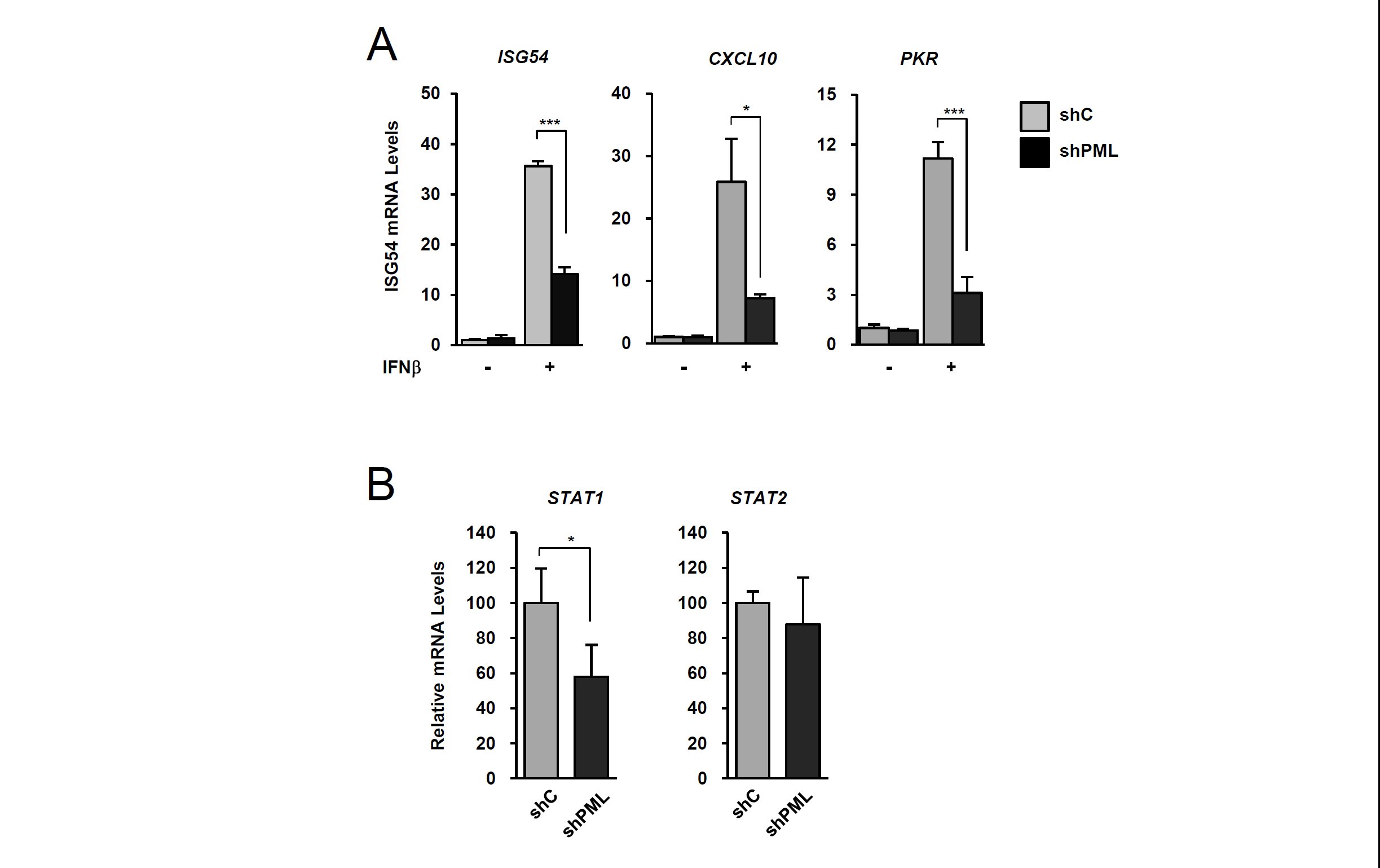

Supplement: S2 Fig — (A) Control (shC) and PML-knockdown (shPML) 293 cells produced using retroviral vectors were untreated or treated with IFNβ (1 x 10^3^ units/ ml) and the mRNA levels of ISG54, CXCL10, and PKR were measured by qRT-PCR. (B) The mRNA levels of STAT1 and STAT2 in control (shC) and PML-knockdown (shPML) 293 cells were measured by qRT-PCR. (TIF) [file ppat.1004785.s002.tif]

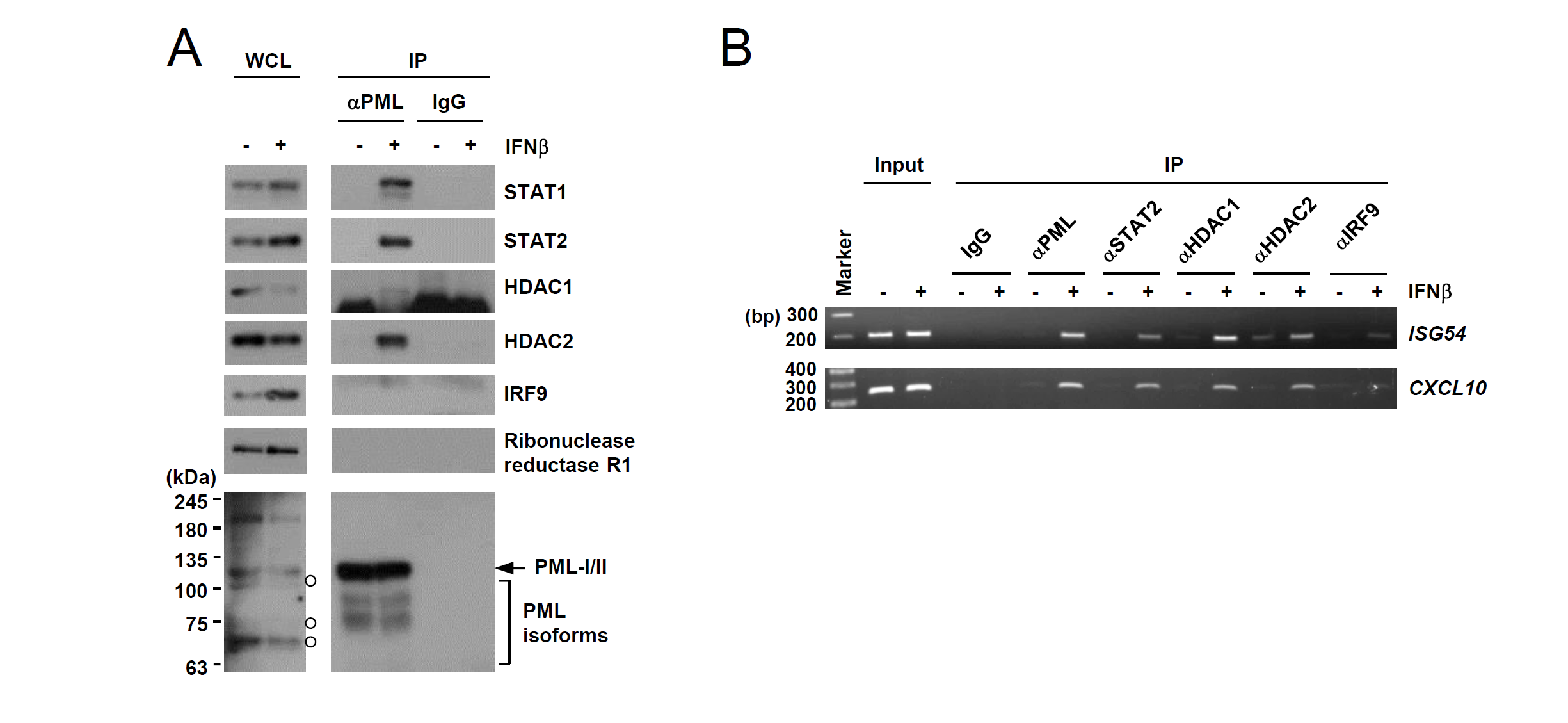

Supplement: S3 Fig — (A) Normal HF cells were treated or not with IFNβ (1 x 10^3^ units/ ml) for 8 h and co-IP assays were carried out. Total cell lysates were prepared and immunoprecipitated with anti-PML antibody (PG-M3) or mouse IgG as a negative control. Immunoprecipitated samples and whole cell lysates were subjected to SDS-PAGE and then immunoblotted with antibodies for STAT1, STAT2, HDAC1, HDAC2, IRF9, ribonucleotide reductase R1, and PML (PG-M3). Circles indicate non-specific bands. (B) HF cells were treated or not with IFNβ as described in (A) and ChIP assays were performed using anti-PML (PG-M3), anti-STAT2, anti-HDAC1, and anti-HDAC2 antibodies. PCR was performed to detect ISG54 and CXCL10 promoter DNAs. The sizes of the DNA fragments amplified from the ISG54 and CXCL10 promoter regions were 199 bp and 241 bp, respectively. A 100 bp DNA ladder was used as a size marker. (TIF) [file ppat.1004785.s003.tif]

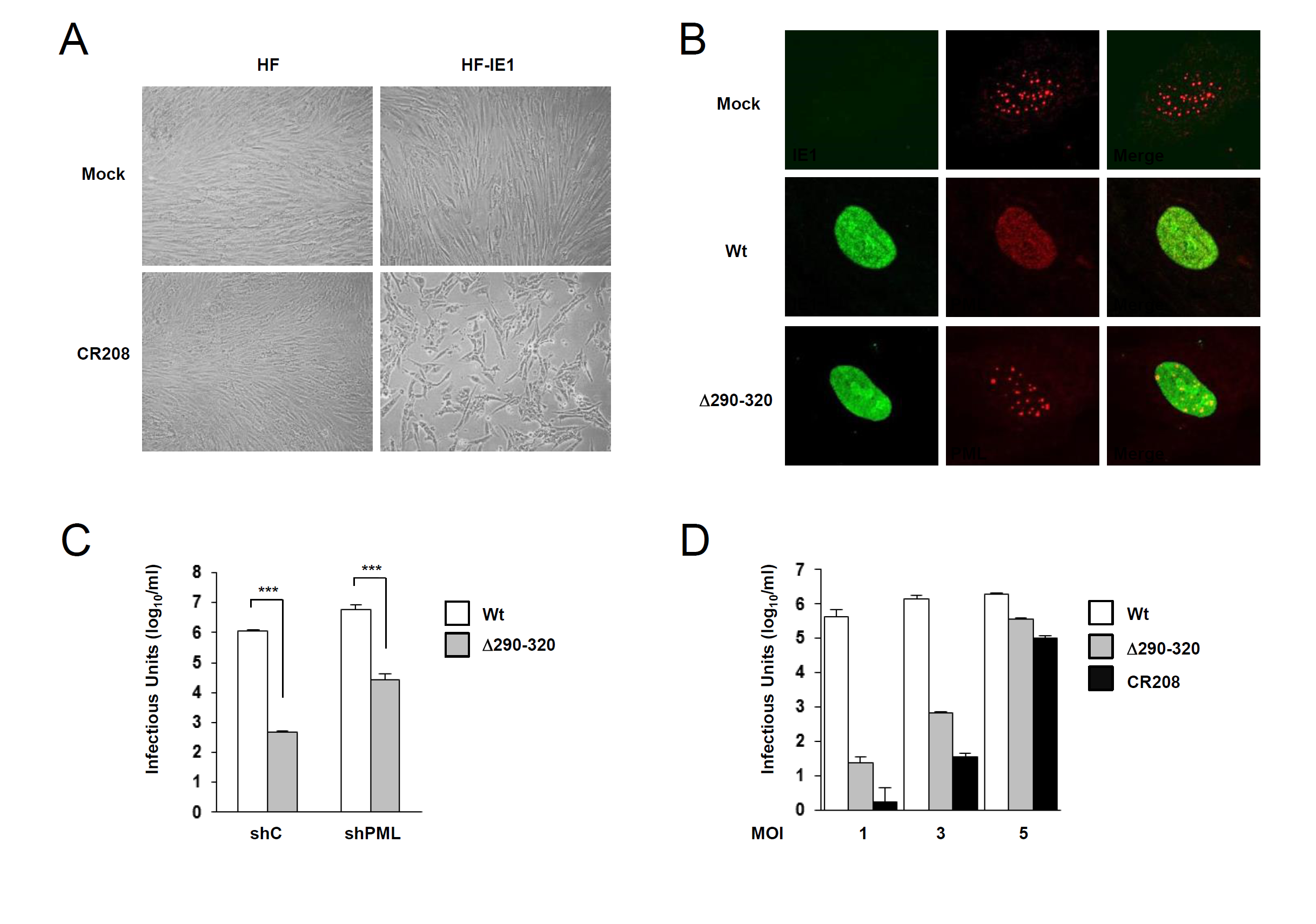

Supplement: S4 Fig — (A) Normal HF and IE1-expressing HF (HF-IE1) cells were mock-infected or infected with CR208 at an MOI of 1 IFU per ml. The phase contrast images were taken at 6 days after infection. The CPE was evident in HF-IE1 cells but not in HF cells after CR208 infection, demonstrating that HF-IE1 cells effectively support the growth of CR208. (B) HF cells were mock-infected were infected with wild-type or IE1(Δ290–320) virus. At 6 h after infection, cells were fixed in methanol and double-label IFA was performed with anti-IE1 (6E1) and anti-PML (PML-C) antibodies. The images were obtained with a Carl Zeiss Axioplan 2 confocal microscope system. (C) shC and shPML HF cells were infected with wild-type or IE1(Δ290–320) mutant virus at an MOI of 3 IFU per cell. At 6 days after infection, the total numbers of infectious units in culture supernatants were determined using infectious center assays. (D) HF cells were infected with wild-type, IE1(Δ290–320) mutant, or CR208 virus at an MOI of 1, 3, or 5 IFU per cell. At 5 days after infection, the total numbers of infectious units in culture supernatants were determined as in (C). (TIF) [file ppat.1004785.s004.tif]

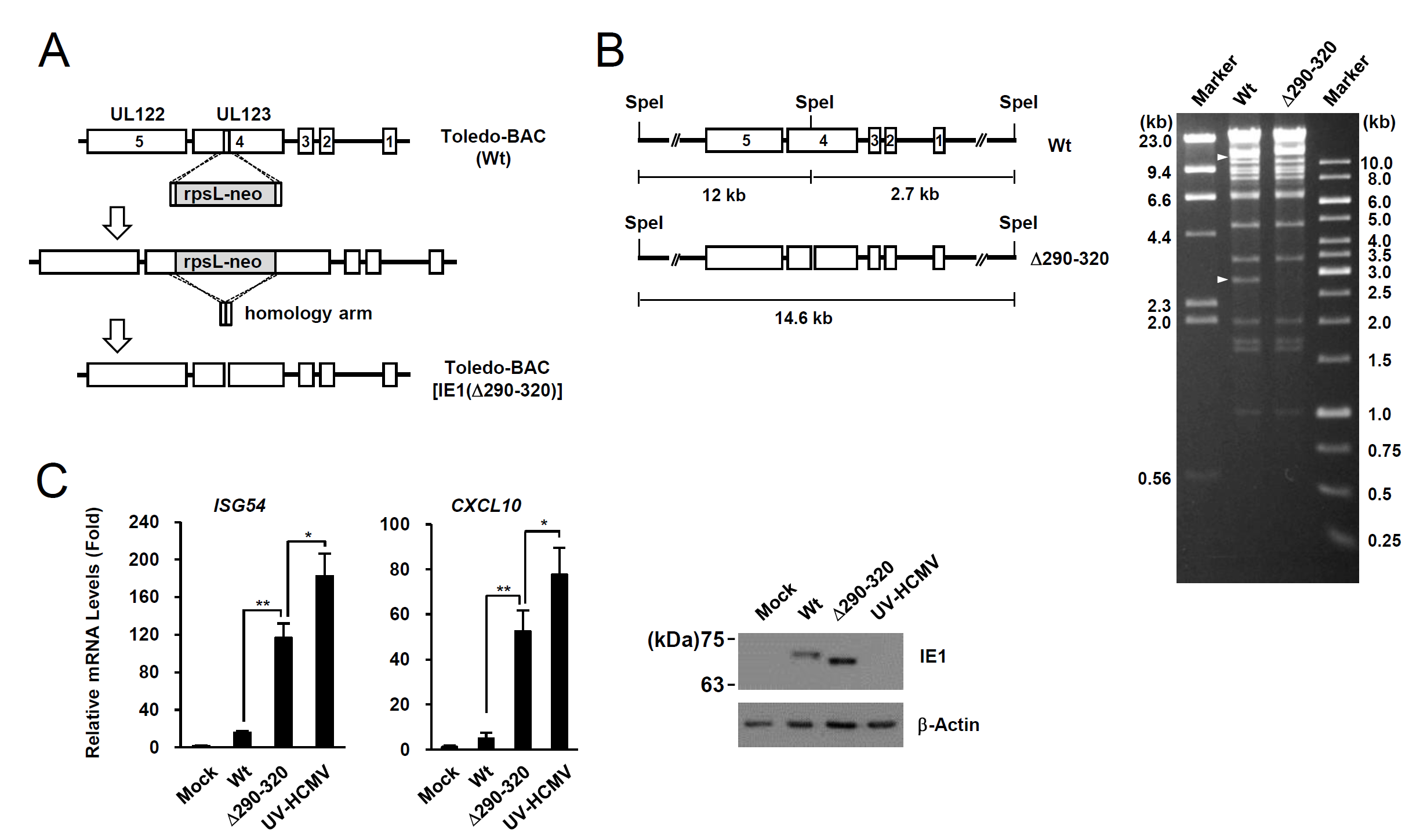

Supplement: S5 Fig — (A) The scheme of the production of a recombinant HCMV (Toledo) virus encoding IE1(Δ290–320). The Toledo-BAC clone was a gift from Hua Zhu (UMDNJ-New Jersey Medical School, Newark, NJ, USA). The Toledo-BAC clone encoding IE1(Δ290–320) protein was produced by using a counter-selection BAC modification kit (Gene Bridges). Briefly, the rpsL-neo cassette DNA was PCR-amplified using LMV1912/1913 primers (see below) containing homology arms consisting of 50 nucleotides upstream and downstream of the target region plus 24 nucleotides homologous to the rpsL-neo cassette. The amplified rpsL-neo fragments with homology arms were purified and introduced into E. coli GS243 containing wild-type Toledo-BAC for recombination by electroporation using a Gene Pulser II (Bio-Rad). The intermediate Toledo-BAC constructs containing the rpsL-neo cassette were selected on Luria Broth (LB) plates containing kanamycin. Next, the rpsL-neo cassette was replaced by annealed oligo DNAs (LMV1914/1915) consisting of only homology arms (50 nucleotides upstream and downstream of the target region). The IE1(Δ290–320) Toledo-BAC was selected on LB plates containing streptomycin. LMV1912; 5’-ATATCCTCACTACATGTGTGGAGACCATGTGCAGTGAGTACAAGGTCACCGGCCTGGTGATGATGGCGGGATCG-3’, LMV1913; 5’-TTGATAACCTCAGGCTTGGTTATCAGAGGCCGCTTGGCCAGCAACACACTTCAGAAGAACTCGTCAAGAAGGCG-3’, LMV1914; 5’-ATATCCTCACTACATGTGTGGAGACCATGTGCAGTGAGTACAAGGTCACCAGTGTGTTGCTGGCCAAGCGGCCTCTGATAACCAAGCCTGAGGTTATCAA-3’, and LMV1915; 5’-TTGATAACCTCAGGCTTGGTTATCAGAGGCCGCTTGGCCAGCAACACACTGGTGACCTTGTACTCACTGCACATGGTCTCCACACATGTAGTGAGGATAT-3’. (B) The wild-type and IE1(Δ290–320) Toledo-BAC clones were digested with SpeI and the pulse-field gel electrophoresis patterns of DNA fragments were shown. The arrowheads indicate the 12 kb and 2.7 kb DNA fragments in the wild-type BAC clone, which disappeared in the IE1(Δ290–320) BAC clone. (C) Recombinant Toledo viruses encoding wild-type IE1 and Δ290–320 mutant were grown in IE1(Towne)-expressing HF cells that [file ppat.1004785.s005.tif]

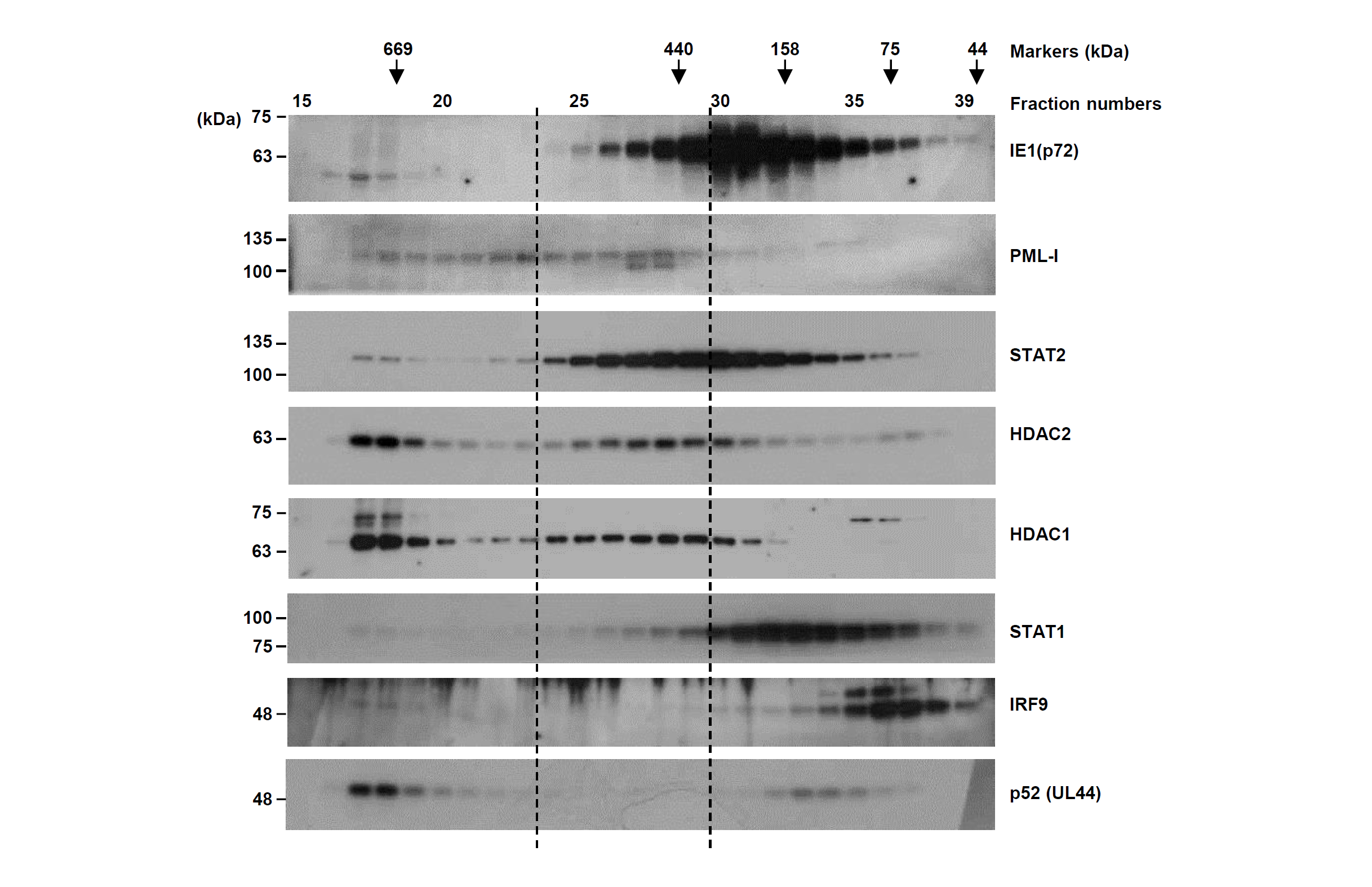

Supplement: S6 Fig — HF cells were infected with HCMV at an MOI of 3 IFU per cell for 24 h. The cell extracts (total 2 mg) were prepared and loaded onto a Superose6 10/300 GL column (GE Healthcare) pre-equilibrated with co-IP buffer. The proteins were eluted at 0.5 ml/min. Each fraction (15 μl) was analyzed by immunoblotting with antibodies for IE1, PML, STAT1, STAT2, HDAC, HDAC2, IRF9 and p52 (encoded by UL44). Apparent molecular mass was evaluated after column calibration with standard proteins [thyroglobulin (669-kDa), ferritin (440-kDa), aldolase (158-kDa), conalbumin (75-kDa), and ovalumin (44-kDa)] in the Gel Filtration Calibration Kit (GE Healthcare). The elution positions of these proteins are indicated at the top. The high molecular mass fractions (>400-kDa), which include IE1, PML, STAT2, HDAC2, and HDAC1, are indicated as dashed lines. (TIF) [file ppat.1004785.s006.tif]

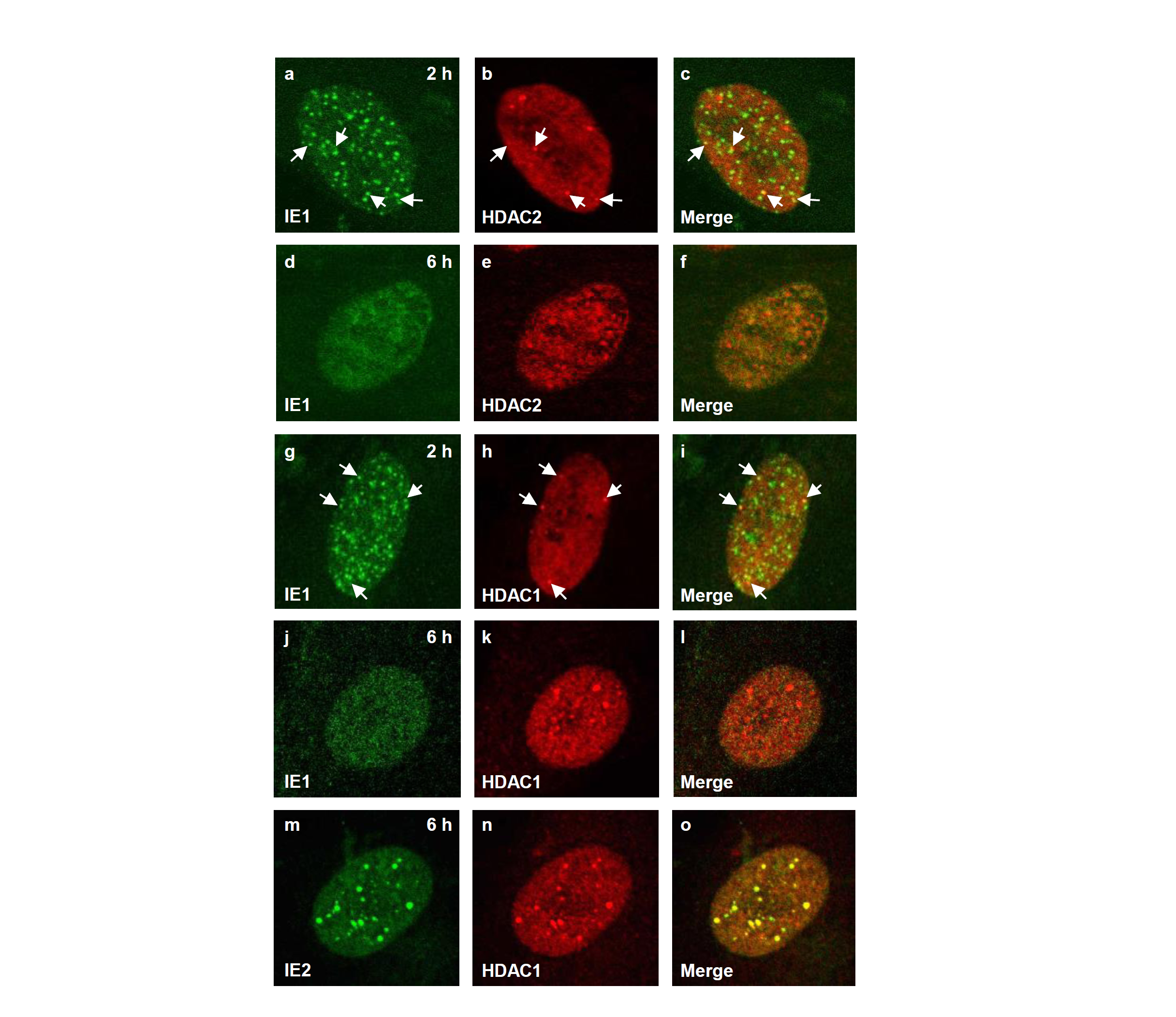

Supplement: S7 Fig — HF cells were infected with HCMV (Towne) at an MOI of 1. Cells were fixed at 2 h (a-c and g-i) or at 6 h (d-f and j-o) after infection in methanol, and confocal double-label IFA was carried out for IE1 and HDAC2 (a-f), for IE1 and HDAC1 (g-l), or for IE2 and HDAC1 as a control (m-o). (TIF) [file ppat.1004785.s007.tif]

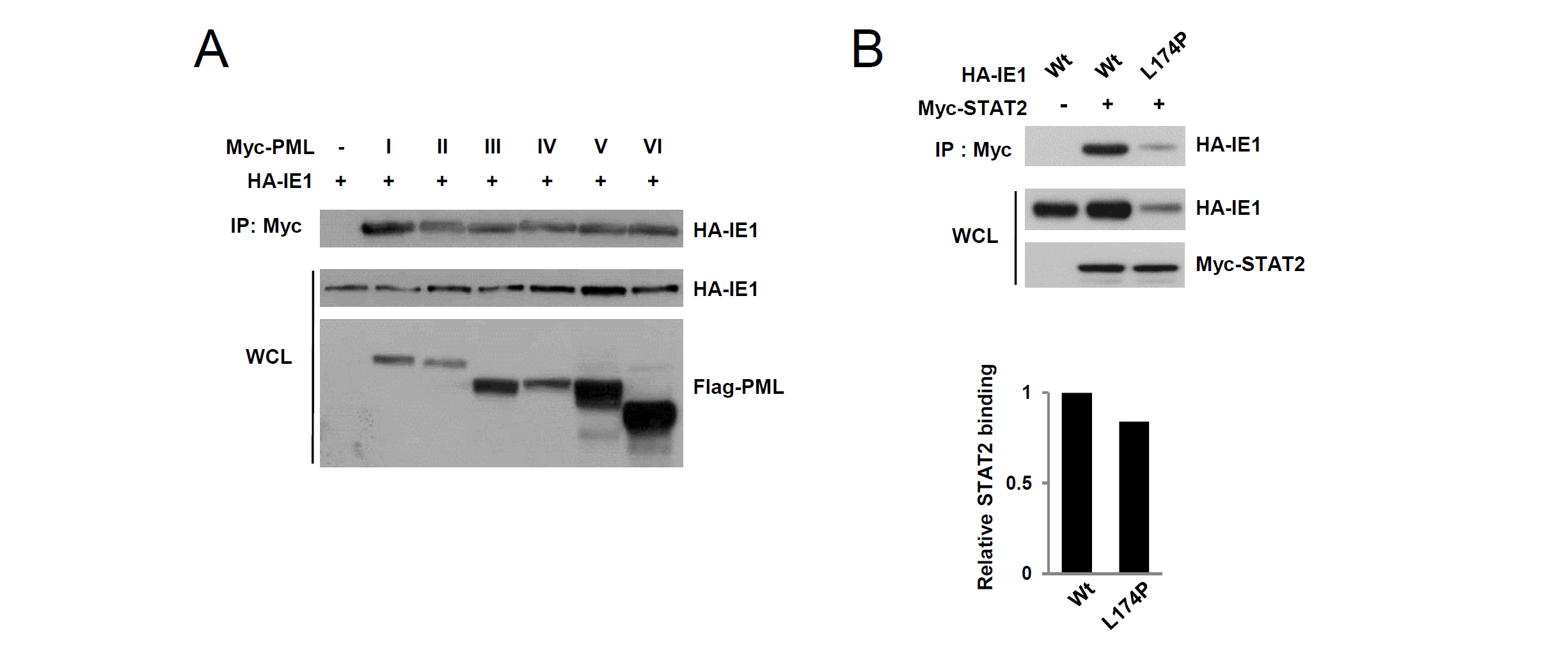

Supplement: S8 Fig — (A) 293T cells were cotransfected with plasmids expressing HA-IE1 and myc-PML isoforms as indicated. At 48 h, total cell lysates were prepared and immunoprecipitated with anti-myc antibody and then immunoblotted with anti-HA antibody. Whole cells lysates were also immunoblotted with anti-HA or anti-myc antibodies. (B) Effect of Leu 174 to Pro substitution within the central hydrophobic region of IE1 on its interaction with STAT2. (Top) 293T cells were cotransfected with plasmids encoding HA-IE1 or HA-IE1(L174P) and myc-STAT2 as indicated. At 48 h, total cell lysates were prepared and immunoprecipitated with anti-myc antibody and then immunoblotted using anti-HA antibody. Whole cell lysates were also immunoblotted with anti-HA or anti-myc antibodies. (Bottom) Bar graph showing the relative amounts of wild-type or L174P mutant IE1 protein bound to myc-STAT2. (TIF) [file ppat.1004785.s008.tif]

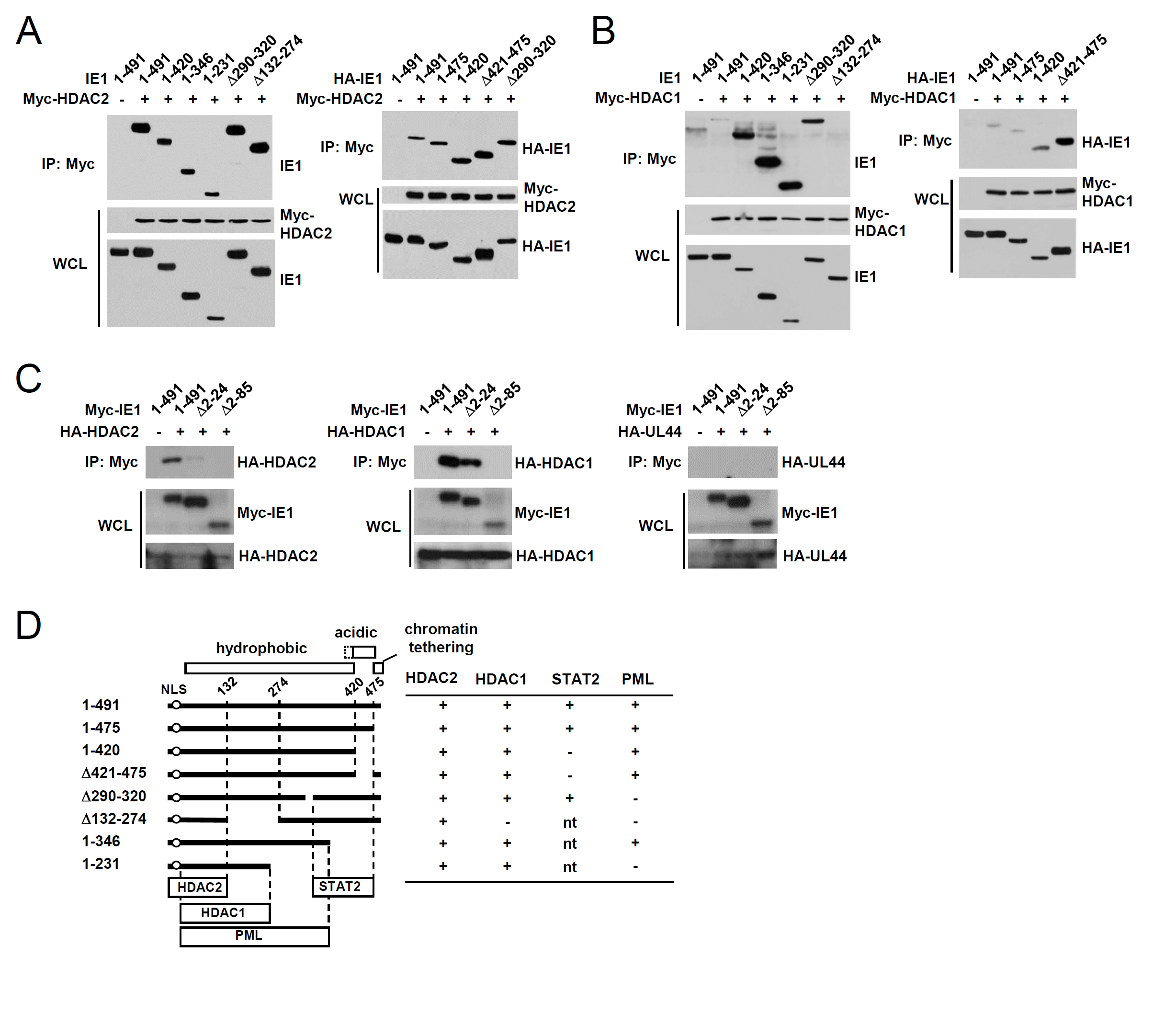

Supplement: S9 Fig — (A) 293T cells were cotransfected with plasmids encoding myc-HDAC2 and untagged wild-type or mutant IE1 proteins (left) or HA-tagged IE1 proteins (right). At 48 h, Co-IP assays were performed using anti-myc antibody and this was followed by immunoblotting with anti-IE1 (6E1) (left) or anti-HA (right) antibodies. The expression levels of IE1 and HDAC2 in whole cell lysates were determined by immunoblotting. (B) Cells were cotransfected with plasmids encoding myc-HDAC1 and untagged or HA-tagged IE1 (wild-type or mutant) proteins as indicated. The Co-IP assay was conducted as described in (A). (C) Cells were cotransfected with plasmids encoding myc-tagged IE1 (wild-type or N-terminal truncated mutants) and HA-tagged HDAC1 or HDAC2 or UL44 (as a control) as indicated. The Co-IP assay was conducted as described in (A). (D) Interactions of wild-type and mutant IE1 proteins with HDAC1, HDAC2, STAT2, and PML are summarized as + (positive interaction) or—(negative interaction). nt: not tested; NLS: nuclear localization signal. The IE1 regions responsible for interactions with HDAC1, HDAC2, PML, and STAT2 are indicated. (TIF) [file ppat.1004785.s009.tif]

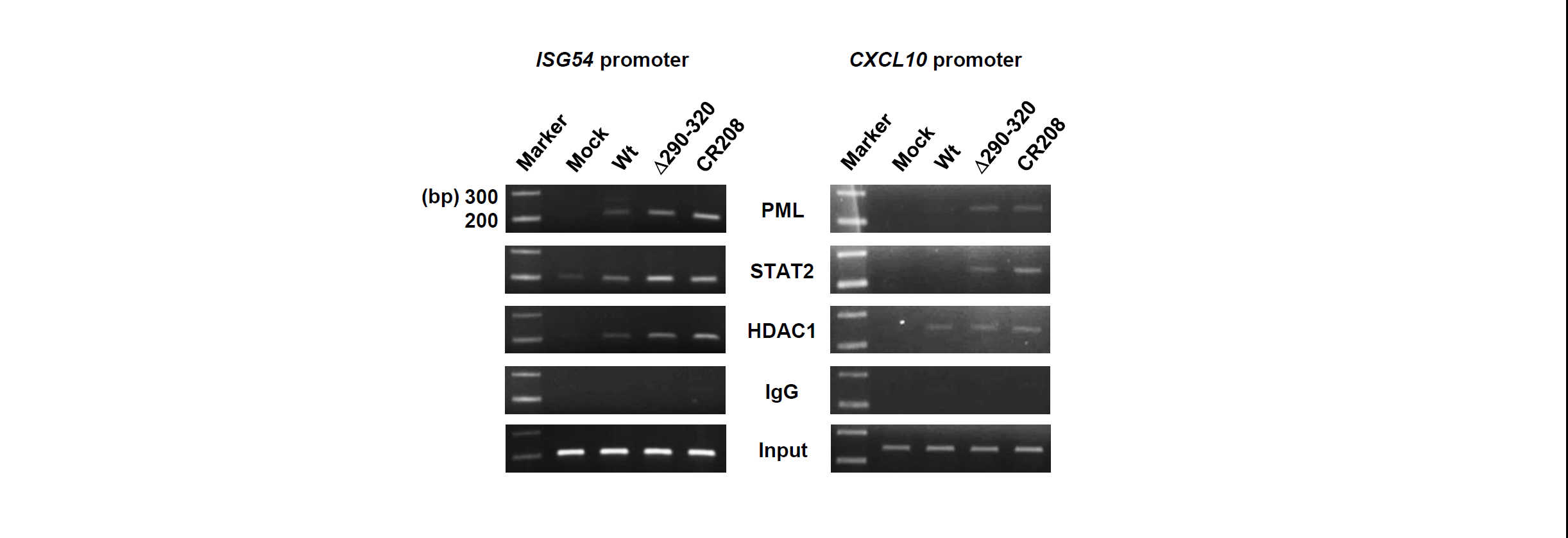

Supplement: S10 Fig — HF cells were mock-infected or infected with wild-type, IE1(Δ290–320), or CR208 virus at an MOI of 3 IFU per cell for 12 h. ChIP assays were performed with anti-PML (PG-M3), anti-STAT2, anti-HDAC1 antibodies or with control IgG to detect the amounts of these proteins bound to ISG54 and CXCL10 promoters. The sizes of DNA fragments amplified from ISG54 and CXCL10 promoters were 199 bp and 241 bp, respectively. The 100 bp DNA ladder size markers are shown. (TIF) [file ppat.1004785.s010.tif]
